# Evolutionary signatures in deep white matter architecture: A comparative study of humans and chimpanzees

**DOI:** 10.1162/IMAG.a.1154

**Published:** 2026-03-11

**Authors:** Maëlig Chauvel, Ivy Uszynski, Marco Pascucci, Yann Leprince, Bastien Herlin, Denis Rivière, Jean-François Mangin, William D. Hopkins, Cyril Poupon

**Affiliations:** BAOBAB, NeuroSpin, Paris-Saclay University, CNRS, CEA, Gif-sur-Yvette, France; Department of Neurophysics, Max Planck Institute for Human Cognitive and Brain Sciences, Leipzig, Germany; UNIACT, NeuroSpin, Université Paris-Saclay, INSERM, CEA, Gif-sur-Yvette, France; Department of Comparative Medicine, Michale E Keeling Center for Comparative Medicine and Research, The University of Texas MD Anderson Cancer Center, Bastrop, TX, United States

**Keywords:** white matter, chimpanzee, fiber clustering, isomap, neuro-evolution

## Abstract

Despite near-identical genetics, humans and chimpanzees display striking cognitive differences, thought to emerge from overall brain size variation and subtle divergences in brain connectivity. We present a comparative analysis of deep white matter bundle (DWMB) morphology in 39 in vivo chimpanzees and 39 humans, using diffusion MRI and a novel isomap-based shape analysis pipeline. After mapping DWMBs into a shared anatomical space via sulcus-informed diffeomorphic registration, we identified robust species-specific differences across key frontal tracts. We focused on four frontal tracts due to their roles in fronto-parietal and fronto-temporal connectivity supporting language, executive function, and socio-emotional processing, with the arcuate fasciculus serving as an internal control given its well-established species differences. Notably, the arcuate fasciculus in humans exhibited greater curvature, volume, and temporal extension—traits absent in chimpanzees and consistent with its role in language. The uncinate and inferior fronto-occipital fasciculus revealed distinct cross-species expansions and lateralization was observed for the frontal aslants and inferior fronto-occipital fasciculus in chimpanzees. These results provide the first high-dimensional morphological mapping of DWMBs across species, uncovering evolutionary adaptations in frontal connectivity and lateralization that likely underlie human-specific cognitive abilities.

## 1 Introduction

Understanding what sets the human brain apart from those of other primates has long been a central question in neuroscience, offering insights into the cognitive and behavioral traits that define our species. Recent advances in magnetic resonance imaging (MRI) have greatly expanded opportunities for comparative neuroanatomy, thanks to the growing availability of high-resolution MRI datasets from both great apes and monkeys (Howard et al., 2023; National Chimpanzee Brain Resource, 2023). Importantly, studies of brain structure and connectivity across species can help infer the evolutionary changes that distinctively underlie human cognitive traits such as complex social learning, language, and tool use (Amiez & Petrides, 2014; Ardila et al., 2016).

While existing resources have enabled the study of large-scale evolutionary changes in the hominid brain (Rilling, 2014), understanding finer-scale differences in brain microstructure and connectivity remains more challenging (Mars et al., 2014). Chimpanzees, who share 99.8% of their genome with humans (Waterson et al., 2005), diverged from our lineage approximately 7 to 8 million years ago (Langergraber et al., 2012). Despite this close genetic relationship, the two species differ in many cognitive and behavioral domains (Mitani et al., 2002), which are reflected in aspects of neuroanatomy (Foubet et al., 2024). Comparative studies have revealed differences in overall brain size and cortical organization (Smaers & Vanier, 2019). Specifically, expansions in both gray and white matter within the frontal lobes have been observed (Preuss, 2011; Smaers et al., 2011)—though not always as expected (Passingham & Smaers, 2014)—along with emerging differences in asymmetry across frontal, parietal, and temporal areas (Gilissen & Hopkins, 2013; Hopkins & Avants, 2013; Schenker et al., 2010). These findings suggest that fronto-parietal and fronto-temporal pathways may have played a central role in structural and functional adaptations unique to the human brain. Accordingly, behavioral capabilities that are highly developed in humans, such as advanced communication, are likely supported by enhanced connectivity within these networks. Within this context, we focused on four major frontal pathways: the arcuate fasciculus (AF), the frontal aslant tract (FAT), the uncinate fasciculus (UF), and the inferior fronto-occipital fasciculus (IFOF). These tracts are implicated in language, motor planning, and socio-emotional cognition (Aron et al., 2007; Geschwind, 1970; Olson et al., 2015). The AF, in particular, has consistently documented inter-species differences and, therefore, serves as an internal control for validating our comparative approach.

Diffusion MRI tractography allows for the reconstruction of white matter architecture in both humans and non-human primates (Basser et al., 2000; Conturo et al., 1999; Poupon et al., 2000). By modeling the directional diffusion of water along axon bundles, tractography enables the identification of deep white matter bundles (DWMBs) that support long-range connectivity. While previous studies have examined DWMBs in humans and chimpanzees using tractography (Bryant et al., 2020; Chauvel et al., 2023), relatively few have offered detailed morphometric characterizations or assessed intra- and inter-species variability (Hecht et al., 2015). These limitations are due in part to the scarcity of high-quality great ape data, as well as the lack of standardized methods to spatially align human and chimpanzee brains for direct comparison.

In this study, we investigated the morphometry of frontal DWMBs implicated in key cognitive functions, including motor control and vocalization, in both humans and chimpanzees. We (1) used in vivo diffusion MRI data acquired with consistent methodologies across species, (2) established a common comparative framework, and (3) developed a robust processing pipeline to minimize interpretive variability and improve reproducibility. Using tractography methods similar to those employed in the Ginkgo Chauvel DWMB chimpanzee atlas (Chauvel et al., 2023), we built a new human DWMB atlas focused on tracts analogous to those previously identified in chimpanzees. Both atlases rely on a fiber clustering approach rather than pre-defined regions of interest, enabling more unbiased comparisons (Guevara et al., 2011, 2012).

Our analyses focused on four frontal tracts: the arcuate fasciculus (AF), the frontal aslant tract (FAT), the uncinate fasciculus (UF), and the inferior fronto-occipital fasciculus (IFOF). The AF and IFOF, in particular, remain the subject of ongoing debate in hominid brain research (Becker et al., 2022; Sarubbo et al., 2019). In humans, the AF links frontal, parietal, and temporal lobes—curving around the posterior lateral fissure from the dorsal premotor cortex to the caudal superior temporal gyrus—and is essential for language processing (Geschwind, 1970). It connects key language regions, including Broca’s and Wernicke’s areas, both of which remain debated in chimpanzee localization. The IFOF links the occipital cortex to inferior frontal areas, passing through the anterior temporal lobe. Once believed to be absent in non-human primates (Petrides & Pandya, 1988; Schmahmann et al., 2009), it has since been identified in macaques (Decramer et al., 2018; Roumazeilles et al., 2020) and chimpanzees (Bryant et al., 2019; Chauvel et al., 2023). The uncinate fasciculus (UF) is a curved white matter tract connecting the anterior temporal lobe, including the amygdala and temporal pole, to the orbitofrontal cortex. It is thought to support the integration of memory, socio-emotional processing, and decision making (Olson et al., 2015; Von Der Heide et al., 2013). While well characterized in humans, its exact functional role and connectivity in non-human primates remain debated, particularly regarding species-specific differences in social cognition. The frontal aslant tract (FAT) links the superior frontal gyrus, including the pre-SMA and SMA, with the ventrolateral inferior frontal gyrus (Broca’s area in humans). This pathway is implicated in motor planning, speech initiation, inhibitory control, and executive functions (Aron et al., 2007; La Corte et al., 2021). Its presence and connectivity in non-human primates have been confirmed more recently (Bryant et al., 2020; Chauvel et al., 2023), but the functional implications in these species remain speculative.

Through this study, we aim to identify quantitative markers of difference in trajectory, connection, and laterality in the DWMBs between the two species and enhance the understanding of the neural architecture that gave rise to human brain specializations. Specifically, we hypothesized that (1) the AF would show greater extension and leftward lateralization in humans than in chimpanzees, reflecting its role in language; (2) the FAT would be more robust and less variable in humans, consistent with its involvement in speech-related motor planning (Aron et al., 2007); (3) the UF would show increased volume and thickness in humans, reflecting enhanced social-cognitive capacities (Von Der Heide et al., 2013); and (4) the IFOF would show species differences in shape and lateralization, possibly related to differences in visual-semantic processing observed in comparative behavioral studies (Andelman-Gur et al., 2020).

## 2 Material and Methods

### 2.1 Chimpanzee cohort and analysis

We considered data coming from 39 healthy *in vivo* chimpanzees including 23 females and 16 males, imaged between 9 and 35 years old (mean = 19 years old) and housed at the Yerkes National Primate Research Center (YNPRC, Atlanta). Chimpanzee MRI scans were obtained from a data archive of scans acquired prior to the 2015 implementation of US Fish and Wildlife Service and National Institutes of Health regulations governing research with chimpanzees. All the scans reported in this publication were completed by the end of 2012 and have been used in previous studies (e.g., Bryant et al., 2020; Chauvel et al., 2023; Vickery et al., 2020). More specifically, the standard MR imaging procedures for chimpanzees at the YNPRC are designed to minimize stress for the subjects, who were scanned during their scheduled physical examination surveys and anesthetized with propofol (40–60 mg/(kg/h)).

Each individual was scanned using a 3 Tesla Trio MRI system (Siemens, Erlangen) with a birdcage coil and a dedicated imaging protocol comprising anatomical (0.625 mm isotropic spatial resolution) and diffusion data (1.9 mm isotropic spatial resolution, b = 1000 s/mm^2^, 60 diffusion directions) for each subject. All procedures were carried out in accordance with protocols approved by YNPRC and the Emory University Institutional Animal Care and Use Committee.

Anatomical and diffusion MRI data were processed using a Python pipeline dedicated to the chimpanzee and human species using the CEA/NeuroSpin in-house C++ Ginkgo toolbox available at https://framagit.org/cpoupon/gkg.

All T1-weighted images of the 39 chimpanzees’ brains were matched to the Juna.chimp chimpanzee template (template release: https://www.chimpanzeebrain.org/) (Vickery et al., 2020) using diffeomorphic direct and inverse non-linear 3D transformations from each individual brain T1-weighted scan to the Juna.chimp template, computed using the ANTs software (Advanced Normalization Tools) (Avants et al., 2009). After correcting for different artifacts (noise, eddy currents, susceptibility artifacts), individual maps of local orientation distribution functions (ODF) were reconstructed from the corrected diffusion MRI scans using the analytical Q-ball model (Descoteaux et al., 2007). A whole-brain streamline regularized deterministic tractography algorithm (1 seed/voxel, forward step 0.4 mm, aperture angle 30°, lower GFA threshold = 0.15) (Perrin et al., 2005) was then applied to each ODF map to generate streamlines within a propagation mask, corresponding to the brain and established from the anatomical MRI, yielding the 39 chimpanzees’ individual tractograms composed of several millions of fibers each. Streamlines whose lengths did not fall within the 5 mm–300 mm range were filtered out.

**Fig. 1.**
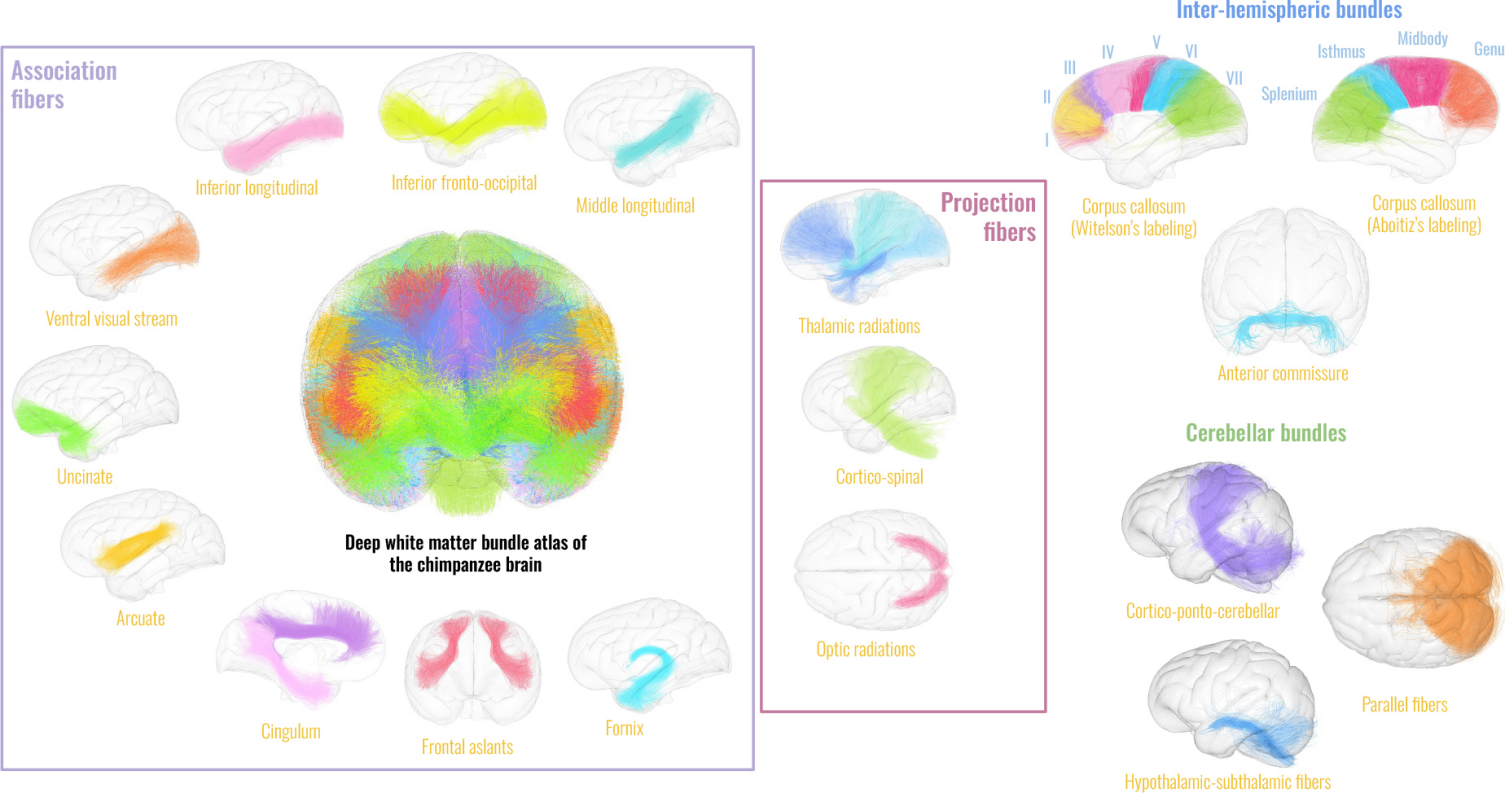
Deep white matter atlas of the chimpanzee brain. Available at https://doi.org/10.5281/zenodo.7147789. Association fibers, projection fibers, inter-hemispheric fibers as well as cerebellar and pontic fibers are displayed on the Juna template cortical pial mesh.

The chimpanzee atlas considered for this study and applied to each subject tractogram is the one described in Chauvel et al. (2023) and available on Zenodo (Chauvel, Uszynski, Herlin, Mangin, et al., 2022), see Figure 1.

### 2.2 Human cohort and analysis

We used a cohort of 39 healthy human subjects from the Human Connectome Project (HCP, release: http://www.humanconnectomeproject.org/) acquired by Washington University in Saint Louis and the University of Minnesota (Van Essen et al., 2013) including 23 women and 16 men, to intentionally match the chimpanzee cohort, aged between 22 and 35 years. It included for each subject a series of anatomical and diffusion-weighted MRI (dMRI) sequences performed on a Connectome Skyra 3T MRI system. The T1w acquisition was performed using a 3D MPRAGE sequence, with a 0.7 mm isotropic spatial resolution and TR/TE = 2400/2.14 ms. The multiple-shell dMRI acquisitions were performed using a 2D monopolar pulsed gradient spin-echo (PGSE) single-shot multi-band EPI sequence (multi-band factor of 3, monopolar diffusion gradient pulses, 1.25 mm isotropic spatial resolution, TR/TE = 5500/89.50 ms) over 3 shells at b = 1000/2000/3000 s/mm^2^ along 90 diffusion directions for each shell, and 6 non-diffusion-weighted (b = 0 s/mm^2^) reference images.

Similarly to the method used for chimpanzees, we designed an analysis pipeline for diffusion-weighted MRI data processing using the CEA/NeuroSpin in-house C++ Ginkgo toolbox. Three consecutive steps were performed for each subject: (1) registration of the subject’s brain MRI to a common atlas space (the MNI ICBM 2009c non-linear asymmetric template) with the ANTs toolbox; (2) computation of the diffusion Orientation Distribution Functions (ODF) with the analytical Q-ball model; and (3) computation of a whole-brain tractogram with a regularized deterministic algorithm.

#### 2.2.1 Fiber clustering

The two-step clustering strategy extensively described in Chauvel et al. (2023) was used to build up fascicle clusters relevant at the human population level. In summary, the implemented analysis pipeline inherits the approach proposed in Guevara et al. (2011) and is based on the use of four consecutive steps:

1. Subdivision of individual tractograms into gross regions (left and right hemispheres, inter-hemispheric region, brainstem and cerebellum) and fiber length ranges, followed by a projection of fibers to a density mask for each length range;
2. Thresholding of each density mask to a binary mask corresponding to a specific gross region and length range;
3. Individual-level clustering composed of five steps: (1) k-means definition of parcels in the binary mask corresponding to each range of fiber length stemming from the tractograms; (2) computation of a connectivity matrix between each pair of parcel measuring the number of fibers connecting the two parcels (and being superior to a minimum threshold count); (3) hierarchical clustering of the connectivity matrix bringing out highly connected parcels forming “clusters”; (4) each cluster corresponds to a set of fibers making up a “fascicle”; (5) further splitting of fascicles depicting a fan shape using a watershed algorithm over a density mask of fiber extremities.
4. Population-level clustering composed of two steps: (1) diffeomorphic transformation of each subject’s fascicle map to a common template space (MNI space for humans); (2) computation of fascicle clusters at the group level from the set of all fascicle *centroids* (sparser representation of a fascicle into a single representative fiber) using a density-based spatial clustering algorithm (DBSCAN). This clustering is inferred from the affinity matrix of normalized symmetric mean of mean closest distances between centroids. Each resulting fascicle cluster represents a connection hub present in a large proportion of the subjects, putatively contributing to a target white matter bundle.

This atlas is available on the Zenodo platform (Chauvel, Uszynski, Herlin, & Poupon, 2022).

### 2.3 Comparison of human and chimpanzee DWMBs

**Fig. 2.**
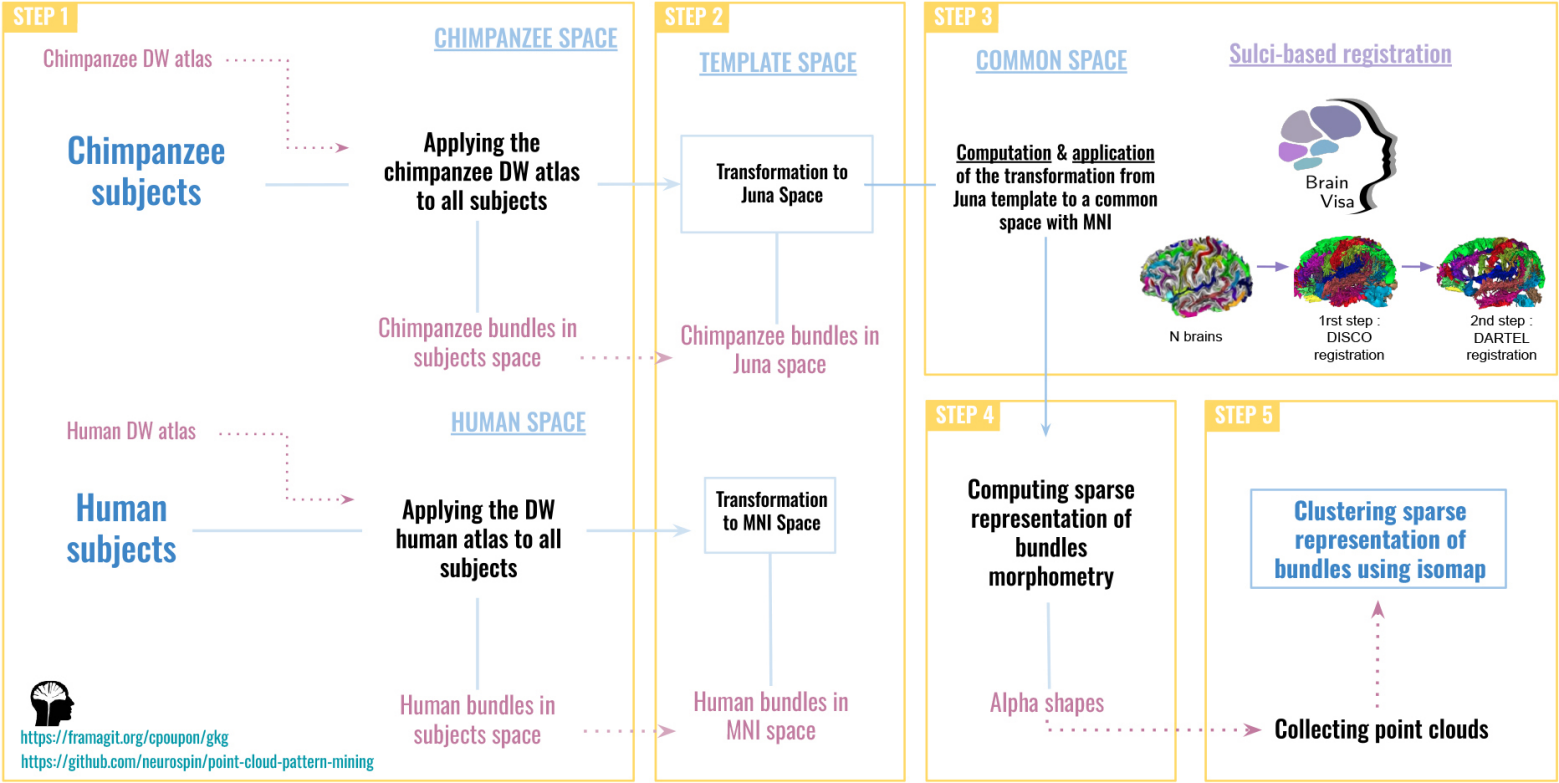
Analysis pipeline. Pipeline of analysis applied on the chimpanzee and human subjects data for the geometrical morphological evaluation of deep white matter bundles.

To analyze the commonalities and differences between human and chimpanzee brain white matter bundle morphologies, similar data processing steps were necessary (see Fig. 2) for the two species:

1. Application of the chimpanzee (/human) DWMB atlas to each cohort’s subject, allowing for the extraction of bundles stemming from the atlas for all subjects.
2. Transformations of each subject’s DWMBs from the subject space to their own species template space.
3. Registration of the MNI/Juna.Chimp template spaces in order to define a common space and transformations of the subjects DWMBs from the template space to this common space.
4. Computation of each subject’s DWMB alpha shapes to get a sparse representation of each DWMB.
5. Application of the isomap algorithm to perform a morphological analysis.

Each of these steps is further detailed below.

#### 2.3.1 Extraction of individual bundles

The human and chimpanzee DWMB atlases were, respectively, applied to each of the 39 subjects of the human and chimpanzee cohorts using the Ginkgo *Advanced Fiber Labeling* tool. The atlases were applied to each subject individually using each subject’s diffeomorphic transformation from the atlas to its own space. The bundle segmentation tool computes for each fiber its pairwise distance to each centroid (sparse representation of a bundle) of the corresponding DWMB atlas and then attributes to the fiber the label of the closest centroid, thus yielding the expected individual DWMB. More details can be found in the SM-1 section, see Supplemenary Fig. S1.

The result of the chimpanzee and human application of the DWMBs on two subjects is shown in the Supplementary Materials (see SM-2 section, Supplementary Figs. S2 and S3).

#### 2.3.2 Transformations of the subjects DWMBs from subjects to the template spaces

All T1-weighted images of the 39 chimpanzees’ brains were matched to the Juna.Chimp chimpanzee template (template release: https://www.chimpanzeebrain.org/) (Vickery et al., 2020) using diffeomorphic direct and inverse non-linear 3D registration transformations computed using the ANTs software (Advanced Normalization Tools) (Avants et al., 2009).

#### 2.3.3 Co-registration of the Juna and MNI templates using a sulcal-based method

Several techniques exist for defining a diffeomorphism between two volumetric brain images, each relying on different constraints and optimization methods. Among the wide variety of available tools for performing such registrations, such as ANTs (Advanced Normalization Tools) (2009), SPM (Statistical Parametric Mapping) (Ashburner, 2007), or FSL registration tool “FNIRT” (FMRIB’s Non-linear Image Registration Tool) (Andersson et al., 2007), those capable of accurately aligning anatomical features are particularly valuable. Indeed, the brain’s surface is characterized by complex folds—sulci and gyri—which play a fundamental role in defining cortical regions. Therefore, registration methods that take into account sulcal patterns are especially useful for aligning anatomically meaningful regions across individuals and across species. This is the motivation behind our choice to use the DISCO (Diffeomorphic Sulcal-based Cortex Organization) registration tool from the BrainVISA/Anatomist software suite (Cointepas et al., 2001), as it specifically incorporates sulcal alignment to guide the diffeomorphic process. The DISCO registration tool was originally introduced in Auzias et al. (2009) to improve diffeomorphic registration of T1-weighted MRI by adding a further constraint in order to more accurately match brain sulci between different individuals. It relies on a twofold process: first, the alignment of the predefined sulci of two T1-weighted MRIs to initialize the diffeomorphism, followed by a second complementary step corresponding to a DARTEL registration (2011).

##### 2.3.3.1 Selection of landmark sulci for the DISCO registration

While the human brain sulci are, for the most part, well known and a developing field of study for the identification of their variability, it is a relatively new field of research for the chimpanzee brain.

**Fig. 3.**
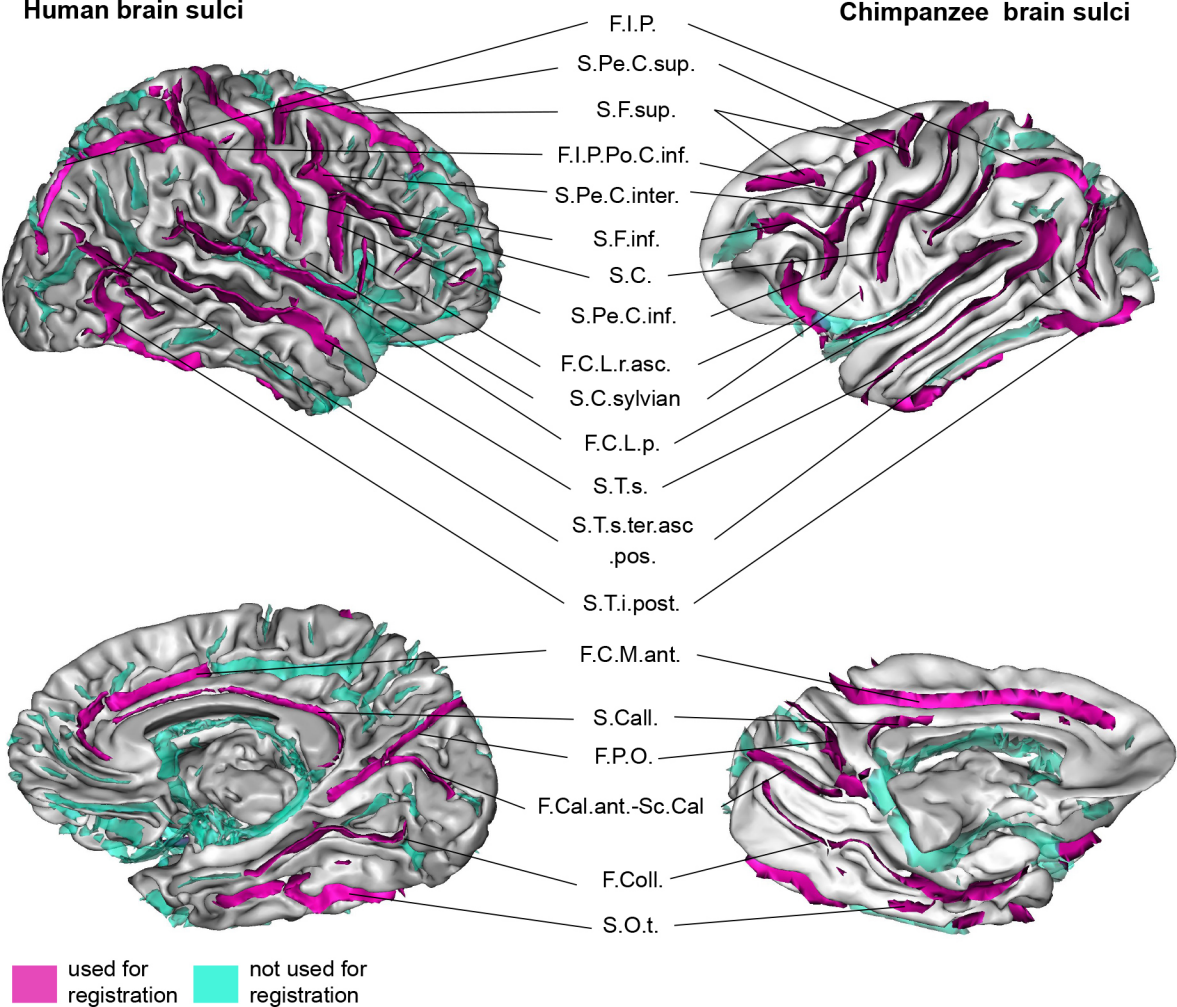
Identification of the brain sulci used as landmarks for the human (MNI) and the chimpanzee (Juna.Chimp) brain templates co-registration. Twenty sulci were identified = F.I.P.: intraparietal fissure, S.Pe.C.sup.: superior precentral sulcus, S.F.sup.: superior frontal sulcus, F.I.P.Po.C.inf.: inferior post-central intraparietal sulcus, S.Pe.C.inter.: intermediate precentral sulcus, S.F.inf.: inferior frontal sulcus, S.C.: central sulcus, S.Pe.C.inf.: inferior precentral sulcus, F.C.L.r.asc.: ascending ramus of the lateral fissure, S.C.sylvian: central sylvian sulcus, F.C.L.p.: posterior lateral fissure, S.T.s.ter.asc.pos.: posterior terminal ascending branch of the superior temporal sulcus, S.T.i.post.: posterior inferior temporal sulcus, F.C.M.ant.: calloso-marginal anterior fissure, S.Call.: subcallosal sulcus, F.P.O.: parieto-occipital fissure, F.Cal.ant.-Sc.Cal: calcarine fissure, F.Coll.: collateral fissure, S.O.t.: occipito-temporal sulcus.

Taking as reference the sulci nomenclature from the human MNI template, the existing research on chimpanzee brain sulci, and the consensus reached with neuroanatomists and experts in brain sulcation (J.-F.M. and Y.L.), the Juna.Chimp template was specifically processed using the BrainVISA/Morphologist toolbox (Mangin et al., 2004) to segment its sulci and label them according to this consensus (Rivière et al., 2022). This cross-species sulci matching then facilitates the co-registration of human and chimpanzee brains using the DISCO-DARTEL tool available in BrainVISA (Rivière et al., 2011). In total, 39 sulci were identified in the chimpanzee brain, of which 20, common with humans, were selected for the brain co-registration, as shown in Figure 3.

##### 2.3.3.2 DISCO and DARTEL co-registration

The DISCO pipeline from the BrainVISA toolbox uses brain sulci to perform an initial diffeomorphic registration step between the two brains to match their homologous sulci, followed by a second step using the well-known DARTEL toolbox. A preliminary pre-processing step involves selecting the corresponding sulci from the chimpanzee and human templates. The success of this step is of utmost importance, as the outcome of the DISCO pipeline directly depends on it. This step proved to be quite challenging: some well-identified sulci in both species, such as the superior temporal sulcus (STS) or the central sulcus (CS), were relatively straightforward to name. However, for other sulci, such as the lunate sulcus in chimpanzees or the frontal sulci in both species, significant variability and deformations in the corresponding cortical areas were observed.

It should be noted that, while the DISCO-DARTEL registration ensures approximate alignment of homologous sulci across species, the subsequent PCA-based alignment performed prior to isomap embedding operates independently of sulcal landmarks. This two-step approach allows the sulcus-based registration to guide the initial anatomical alignment without imposing constraints on the morphometric analysis, which is ultimately driven by point cloud geometry. Thus, the PCA step ensures that morphological comparisons are primarily sensitive to bundle shape rather than local sulcal variation.

#### 2.3.4 Computation of a sparse representation of DWMBs using alpha shapes

Once the atlas is applied and the bundles are retrieved from all subjects, the amount of information is colossal, with millions of streamlines. Each streamline is defined by so many points that the resulting point cloud becomes too large to be processed using the isomap algorithm. To overcome this limitation, a sparse representation of each DWMB was computed relying on a simple alpha shape of the point cloud composed of all the points belonging to all the streamlines composing the bundle.

**Fig. 4.**
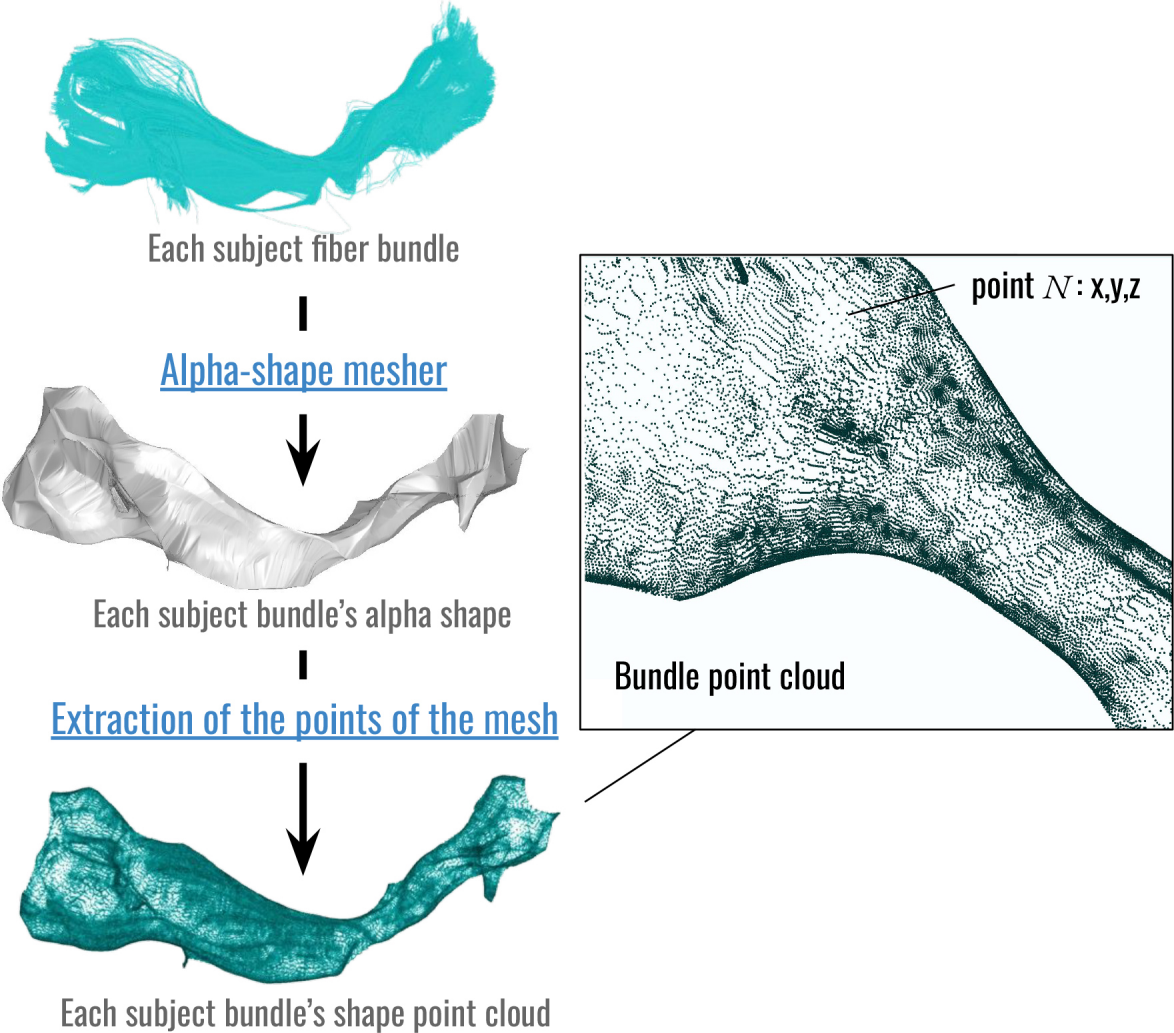
Sparse representation of each DWMB using alpha shapes. This alpha shape is computed from all the points belonging to all the streamlines composing the bundle and represents the surface envelope of the white matter bundle.

The alpha shape represents the surface envelope of the white matter bundle, significantly reducing its representation to a few hundred points instead of thousand points (see examples on Fig. 4).

To reduce the influence of peripheral streamlines, we applied a density-based cleaning step before surface reconstruction, ensuring that only the coherent core of each bundle contributed to the final point cloud. This procedure minimizes bias introduced by isolated or noisy streamlines at the periphery of the tract. As a result, the reconstructed surface and subsequent isomap embedding primarily reflect global morphological variations rather than local termination variability.

#### 2.3.5 Morphological analysis using isomaps

**Fig. 5.**
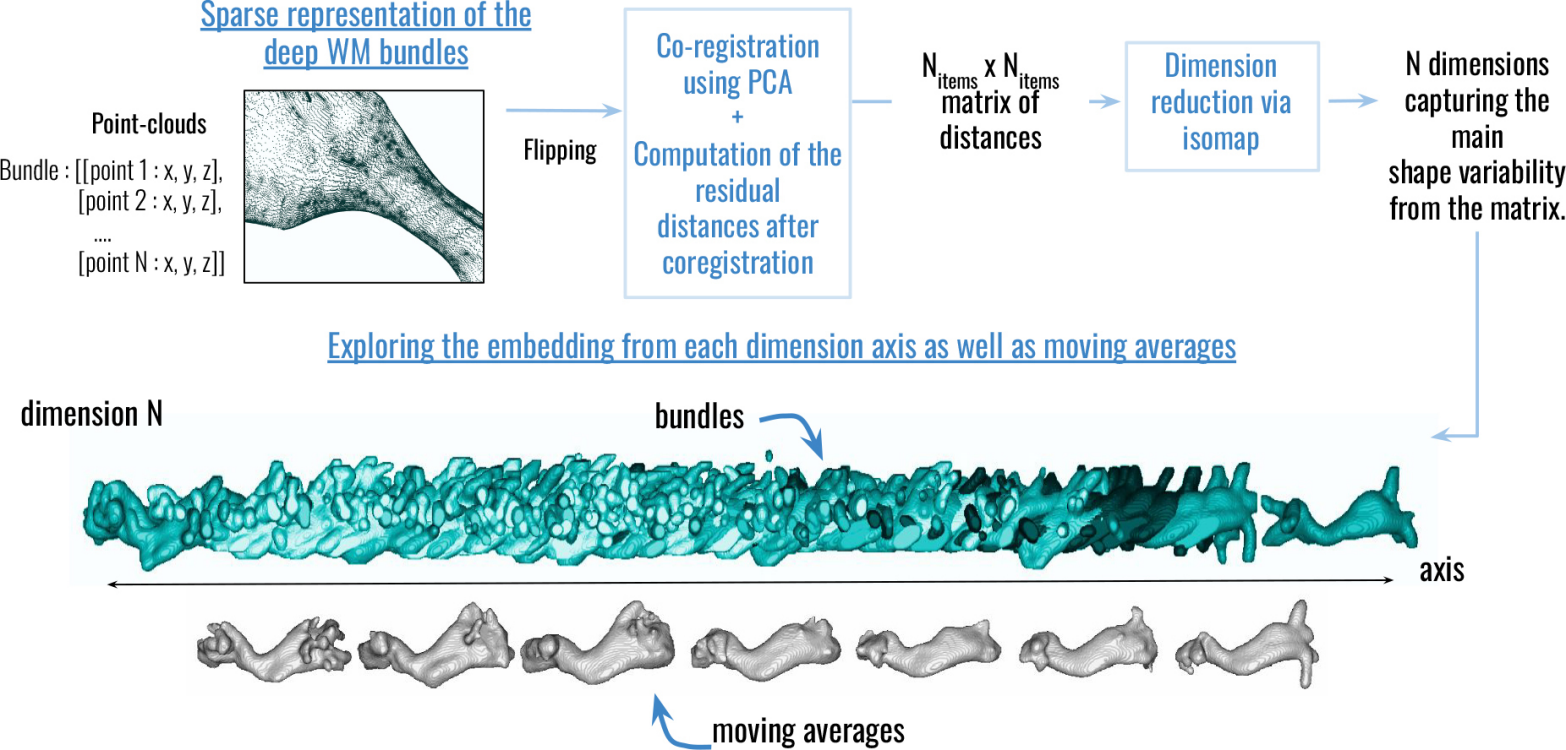
Analysis pipeline from bundle point clouds to shape variability along a certain embedding dimension. Point clouds from the considered bundle’s alpha shape are flipped from the left hemisphere to the right, then co-registered using PCA to obtain residual distances stored in a matrix. This matrix is then subjected to dimensionality reduction using isomap to capture the main shape variability, which can be analyzed across the different resulting dimensions of the embedding.

This chosen approach, using point clouds as inputs, aims to represent or capture shape variability between different objects, by the computation of the point distances and the application of a dimension reduction (Tenenbaum et al., 2000). This method, inspired from Sun et al. (2017) and already applied to study SWMB shapes between humans and chimpanzees (Chauvel et al., 2024), relies on point cloud analysis (see Fig. 5).

In both the human and chimpanzee brains, the sparser alpha shape-based representation of DWMBs resulted in a straightforward point cloud description for each DWMB. This point cloud representation serves as an efficient means to analyze shape variations among DWMBs while retaining the key characteristics of their shapes.

Before clustering DWMB shapes using the isomap algorithm, their point cloud representations were re-aligned into a common space using a Principal Component Analysis (PCA) approach. This initial step involved extracting the three primary axes of each point cloud and aligning these principal directions across all point clouds through the application of optimal affine transformation matrices (Bellekens et al., 2014).

The geometrical distance between two bundles A and B was then calculated using the symmetric point-to-point pairwise distance between A and B, two bundles represented by $N_{p}$ control points $\left\{P_{A}\left(i\right)\right\}$ and $\left\{P_{B}\left(i\right)\right\}$:

$$d_{pairwise}(A,B)=min\left(\sqrt{\sum_{i=0}^{N_{p}-1}{\left(P_{A}\left(i\right)-P_{B}\left(i\right)\right)}^{2}},\;\;\sqrt{\sum_{i=0}^{N_{p}-1}{\left(P_{A}\left(i\right)-P_{B}\left(N_{p}-i\right)\right)}^{2}}\right).$$ (1)

All the distances between all bundles were stored in a symmetric matrix.

Our clustering strategy was twofold: first, the isomap algorithm was applied to reduce the D-dimensional space as described in Tenenbaum et al. (2000). Its number-of-neighbors parameter was empirically chosen at a value of 6. Next, we proceeded with the exploration of each dimension of the embedding. Each dimension orders the bundles according to specific morphological features, which are highlighted by the calculation of sliding averages. In the study presented here, we did not go further than D = 6.

The employed clustering pipeline is derived from a Python module, as detailed in Pascucci et al. (2022).

This methodology was applied to both chimpanzee and human species separately to first explore possible differences in laterality of the bundles and it was also concomitantly applied to both species registered in their common space to identify cross-species differences.

To assess whether there is a significant difference in the morphologies of brain bundles between the left and right hemispheres within and between species, we performed an independent two-sample t-test after verification for normality (Shapiro) and homogeneity of variance (Levene).

Statistical analyses were performed using Python. We conducted independent two-sample t-tests to evaluate differences between species, reporting p-values and effect sizes for each analysis. The significance threshold was set at p < 0.05. Sample sizes for each analysis are detailed in the figure legends (Boxes = 25/75% percentile; lines = median; whisker = most extreme data value).

To account for multiple statistical tests across embedding dimensions and bundles, we applied a false discovery rate (FDR) correction using the Benjamini–Hochberg procedure (Benjamini & Hochberg, 1995), see Supplementary Materials (SM-5 section) for details.

## 3 Results

### 3.1 A novel atlas of the human DWMBs homologous to the chimpanzee DWMB atlas

**Fig. 6.**
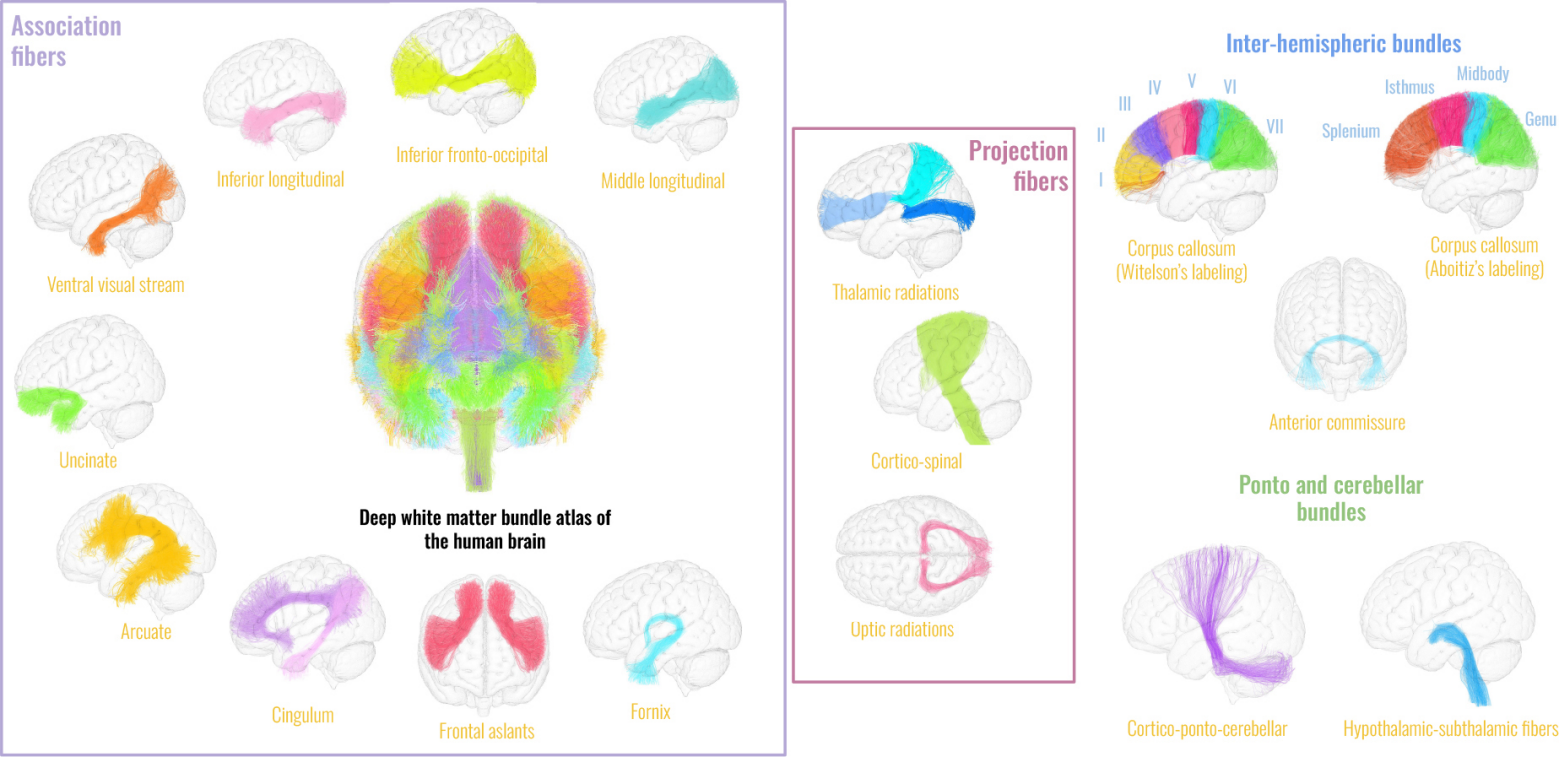
Deep white matter atlas of the human brain. Available at https://doi.org/10.5281/zenodo.7308510, containing 39 bundles specifically targeted to match the chimpanzee deep white matter bundle atlas. Association fibers, projection fibers, inter-hemispheric fibers, as well as cerebellar and pontic fibers are displayed on the MNI template cortical pial mesh.

The description of the human atlas used and entirely designed for this study called the Ginkgo Chauvel human atlas are given in Chauvel (2023). A representation of its DWMBs is shown in Figure 6.

This atlas is composed of 39 white matter bundles voluntarily matching the ones of the chimpanzee including:

- symmetrically on both hemispheres, the anterior, superior, and posterior thalamic radiations (optic radiations), the arcuate, dorsal, and ventral cingulum, the cortico-spinal tract, the fornix, the frontal aslants, the inferior fronto-occipital fascicle, the inferior longitudinal fasciculus, the middle longitudinal fascicle, the optic radiations, the uncinate fascicle, and the visual occipito-temporal fibers (also called ventral visual stream);
- inter-hemispheric bundles such as the anterior commissure and the corpus callosum containing Witelson’s and Aboitiz’s subdivisions;
- cerebellar and pontic bundles, such as the hypothalamic–subthalamic fibers and the cortico-ponto-cerebellar fibers.

Explanations regarding the validation of the tracts of interest in this study are provided in Supplementary Materials (SM-3 section, see Supplementary Figs. S4 to S7).

### 3.2 Species templates co-registration

The registration of the two brain templates showed good results, at the exception of the most anterior part of the inferior frontal cortex. However, it remains obviously complex to register a brain area that is barely present in the chimpanzee brain, which is why this issue was expected but considered as non-impacting for the rest of the analysis, for more details see the Supplementary Materials (SM-4 section, Supplementary Fig. S8).

**Fig. 7.**
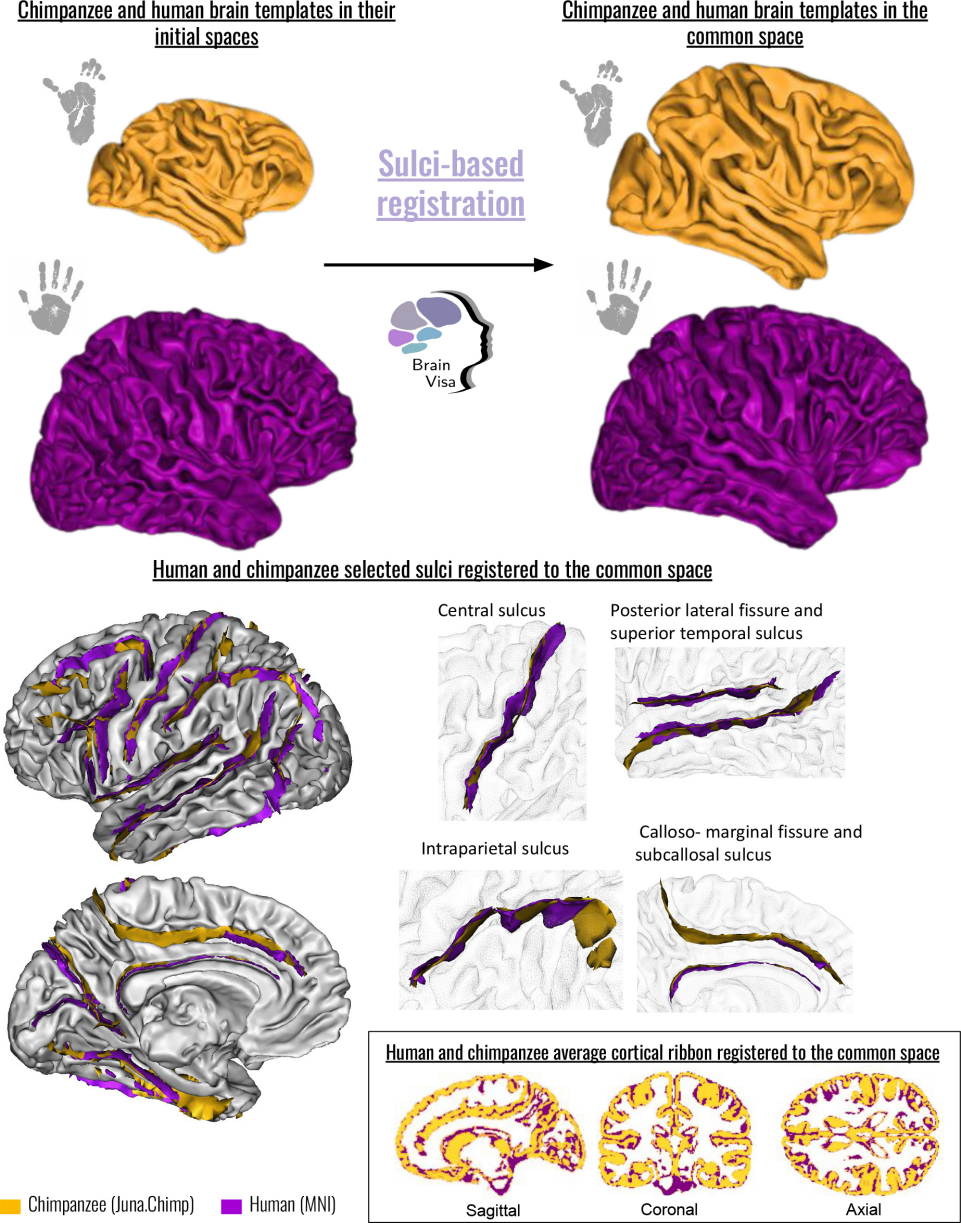
Results of the co-registration between the chimpanzee and human brain templates. Top: Average brain templates in their local space and in the DISCO-DARTEL common space. Bottom: Results of the registration of the selected sulci after DISCO and DARTEL transformations to the common space, as well as some examples of major sulci that guided the inter-species co-registration. Bottom right hand corner: Human and chimpanzee average cortical ribbon registered to the common space.

The registration of the two brain templates was based on 20 sulci and the resulting registration results are shown in Figure 7.

The results of these registrations are given at https://doi.org/10.5281/zenodo.13935442, including transformations and results from the Juna.Chimp template space to the MNI template space.

### 3.3 DWMB morphological analysis

Morphometric differences between bundles were primarily assessed using isomap embeddings, which capture high-dimensional shape variation. While traditional statistical tests can be applied to specific dimensions of the embedding (e.g., differences in the first or second principal dimensions), some aspects of bundle variability are inherently complex and not fully reducible to univariate statistics. Consequently, our approach emphasizes both statistically significant differences in individual dimensions and complex morphological patterns that are observable across the high-dimensional embedding. All reported results survived FDR-corrected p < 0.05 (see SM-5 section, Supplementary Table S1).

#### 3.3.1 The arcuate fasciculus (AF)

The arcuate fasciculus (AF) is commonly described as a curved white matter bundle that connects the inferior frontal gyrus, extending caudally to the temporo-parietal junction, to the inferior temporal cortex (Bernard et al., 2019). This bundle is typically described as connecting two major language-related regions: Broca’s area in the inferior frontal gyrus (speech production region) and Wernicke’s area (speech comprehension region) in the posterior superior temporal gyrus (Geschwind, 1970). It is often divided into one long (direct) segment connecting the inferior frontal gyrus and the temporal lobe, and two short (indirect) segments: an anterior (or dorsal) segment connecting the frontal and parietal lobes and a posterior (or ventral) segment connecting the parietal and temporal lobes (Catani et al., 2005). The AF is a focal point of interest in the scientific community due to its neural composition, structural properties, and functional implications.

An equivalent of this bundle has previously been described in the non-human primate brain (Bryant et al., 2020; Chauvel et al., 2023; Eichner et al., 2024; Frey et al., 2014; Rilling et al., 2008), where speech production is absent.

**Fig. 8.**
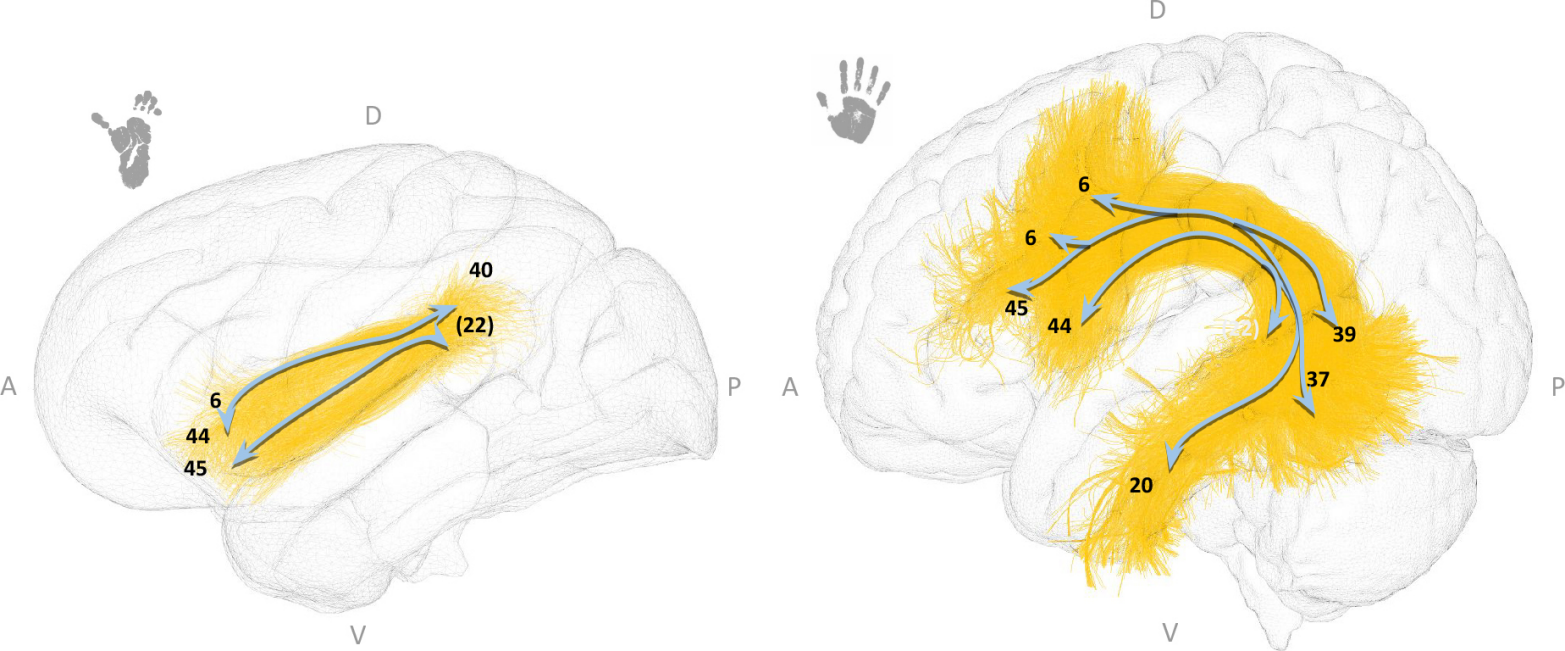
The arcuate fasciculus in chimpanzees (left) and humans (right). Both species’ bundles are displayed in their respective templates (Juna.Chimp for chimpanzees and MNI for humans). Anatomical orientation labels are indicated as A (anterior), P (posterior), D (dorsal), and V (ventral). Numbers correspond to the Brodmann areas reached by the bundle extremities, while numbers in parentheses indicate regions reached by only a small proportion of streamlines. Blue arrows are schematic visual aids highlighting the main bundle trajectories and cortical targets reached by each bundle extremity.

The arcuate fasciculus exhibits considerable differences between humans and chimpanzees (see Fig. 8). In chimpanzees, the AF displays a curved trajectory with fibers originating from the inferior frontal gyrus and extending caudally to the temporo-parietal junction, but it lacks a distinct ventral component. In this study, the chimpanzee AF seemed to connect Brodmann areas (BAs) 44, 6 and to some extent BA 45 in the frontal cortex with BA 40. Some fibers tend to reach BA 22. In contrast, in humans, the AF seemed more robust and displayed a prominent ventral component. The human AF was thicker, larger, connecting BAs 44, 45, 6 in the frontal cortex to BAs 39 and 37, and also exhibiting a ventral trajectory that extends into the temporal lobe to reach the inferior temporal gyrus (BA20).

##### 3.3.1.1 Comparative morphology using isomap

**Fig. 9.**
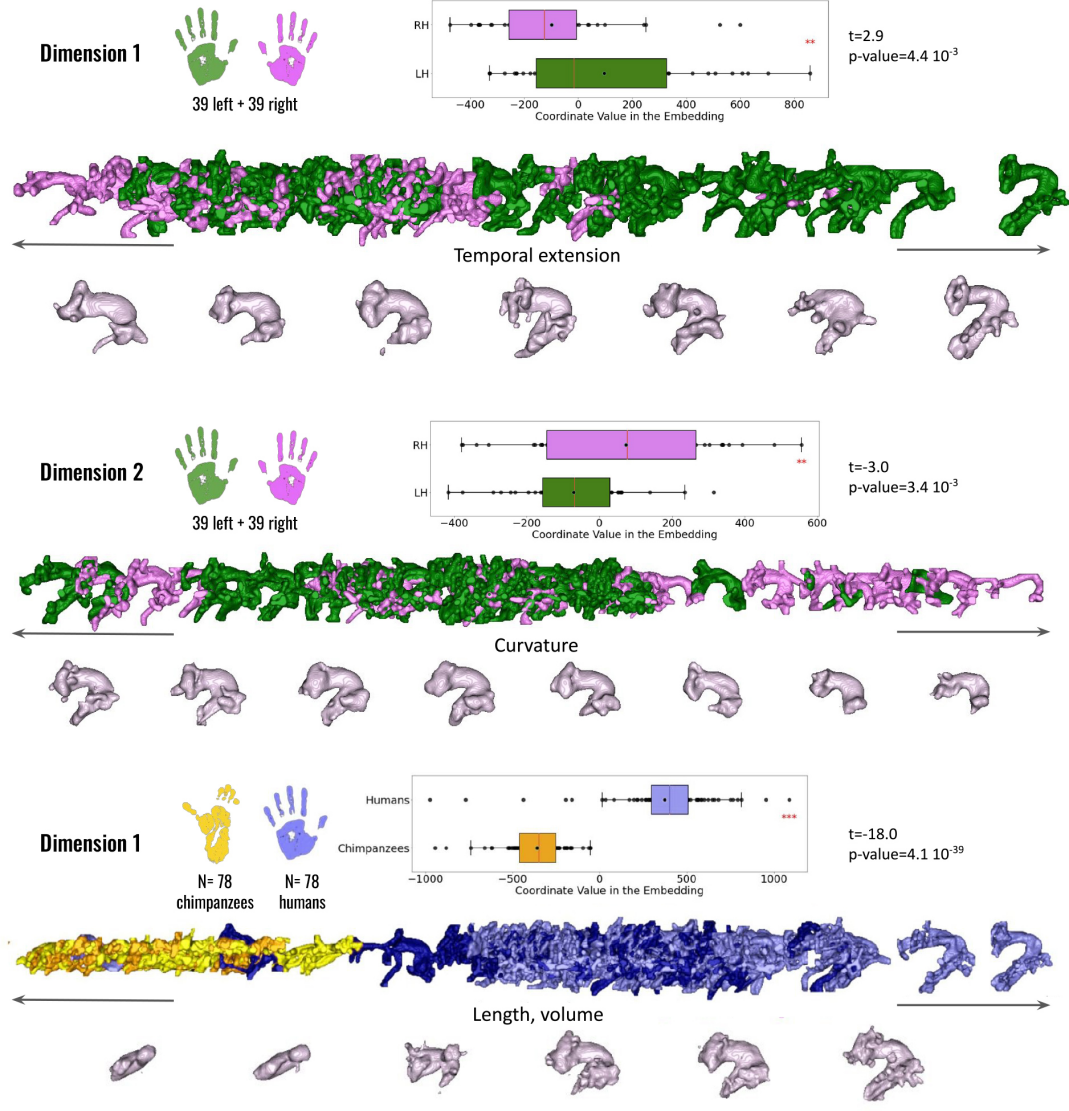
Geometrical analysis of the arcuate fasciculus within humans, chimpanzees, and between species. The first and second dimensions of the isomap reveal significant hemispheric differences in humans (colors: green = left hemisphere; violet = right hemisphere). The first dimension also shows significant interspecies differences between humans and chimpanzees (colors: yellow = chimpanzees; blue = humans). Anatomical and morphological feature labels have been added at the center of each axis to facilitate interpretation of the embedding dimensions.

In humans, two principal dimensions of variability in the arcuate fasciculus (AF) were identified. The first dimension revealed that the shape of the left arcuate fasciculus has a distinct temporal extension, which is predominantly more pronounced in the left hemisphere than the right (paired t-test: t(38) = 2.9, p = $4.4\times 10^{-3}$ , see Fig. 9, first row).

The second dimension also highlighted a difference in bundle shapes between the two hemispheres, though its interpretation is more complex. This dimension appeared to be influenced by the degree of curvature of the fasciculus, with the left arcuate fasciculus showing a more “developed” and “open” shape than the right. Additionally, it seemed to be affected by bundle thickness, appearing more important for the left hemisphere bundles than for the right (paired t-test: t(38) = -3.0, p = $3.4\times 10^{-3}$ , see Fig. 9, second row).

In the chimpanzee brain, no significant differences were observed between the left and right arcuate fasciculus morphologies.

A noticeable geometrical distinction between species was anticipated for the left and right arcuate fasciculus. Visual inspection of the atlas revealed significant shape differences between humans and chimpanzees, and the first dimension of the isomap analysis effectively differentiates the two species (independent t-test: t(76) = -18.0, p = $4.1\times 10^{-39}$ , see Fig. 9, third row).

The key discriminant factors in this comparison are likely the volume and curvature of the bundle. As described in the previous section, the chimpanzee’s arcuate fasciculus extends to the inferior frontal lobe, approximating Broca’s area, and reaches the upper segment of the temporal lobe. This suggests that only the dorsal part of the arcuate fasciculus is present in the chimpanzee brain, in contrast to the more extensive fasciculus observed in humans.

#### 3.3.2 The frontal aslant tracts (FAT)

The frontal aslant tracts (FAT) are relatively short, superficially located white matter pathways within the frontal lobes. They connect the superior frontal gyri to the ventrolateral aspect of the inferior frontal gyri (La Corte et al., 2021). These tracts were recently identified (Catani et al., 2012) and are believed to be involved in various cognitive and motor functions. Research suggests that the FATs contribute to speech and language processing, executive functions, visual-motor coordination, oro-facial movements, inhibitory control, working memory, social behaviors, attention, and music processing (Dick et al., 2019; La Corte et al., 2021).

**Fig. 10.**
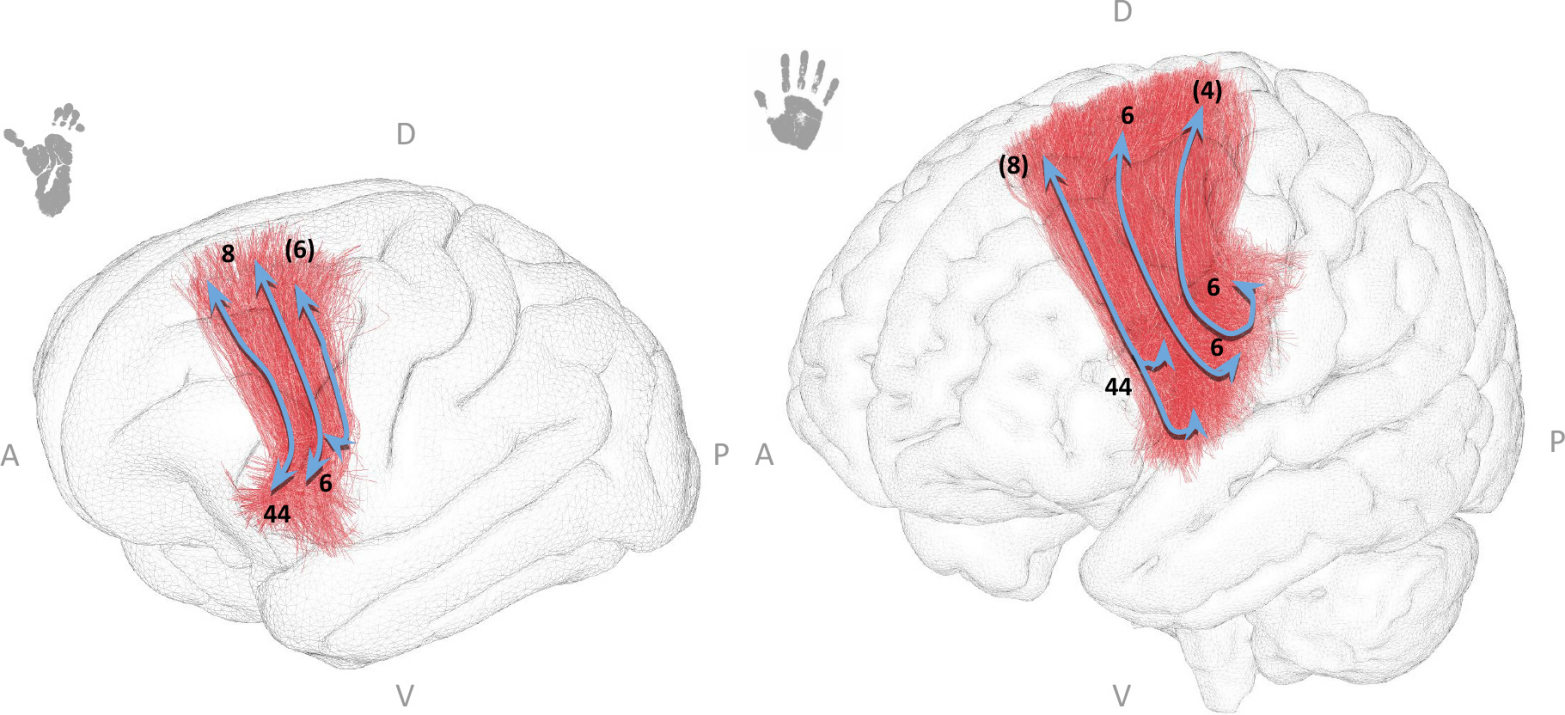
The frontal aslant tract in chimpanzees (left) and humans (right). Both species’ bundles are displayed in their respective templates (Juna.Chimp for chimpanzees and MNI for humans). Anatomical orientation labels are indicated as A (anterior), P (posterior), D (dorsal), and V (ventral). Numbers correspond to the Brodmann areas reached by the bundle extremities, while numbers in parentheses indicate regions reached by only a small proportion of streamlines. Blue arrows are schematic visual aids highlighting the main bundle trajectories and cortical targets reached by each bundle extremity.

In both humans and chimpanzees, the FATs exhibit similar overall connectivity patterns (see Fig. 10), linking Brodmann area 8 of the superior frontal cortex to Brodmann areas 44 and 6 in the inferior frontal cortex. However, visually, the FATs appear to extend more anteriorly into the inferior frontal regions in chimpanzees and are thinner than in humans.

##### 3.3.2.1 Comparative morphology using isomap

**Fig. 11.**
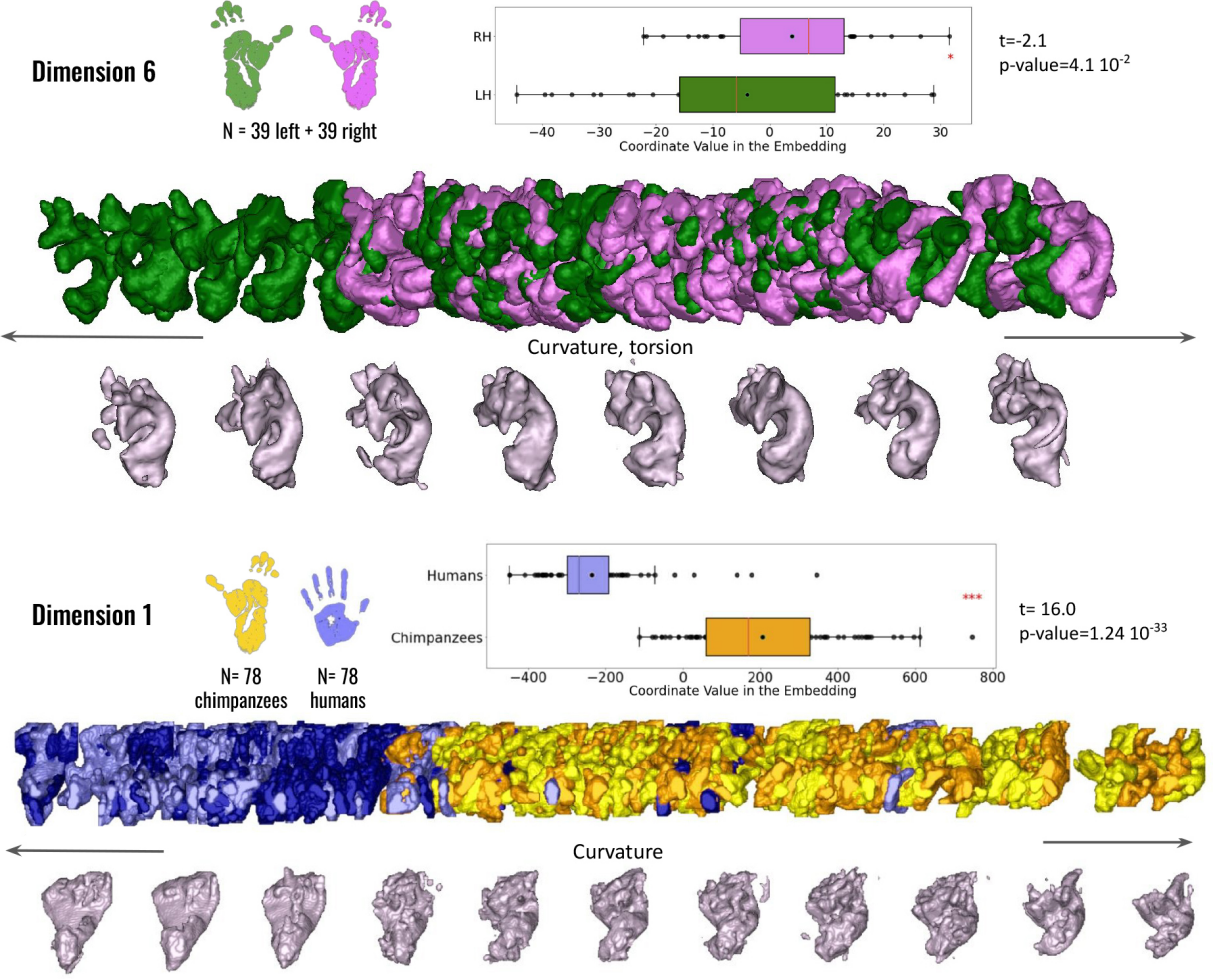
Geometrical analysis of the frontal aslant tracts (FAT) within chimpanzees and between species. The sixth dimension of the isomap reveals significant hemispheric differences within chimpanzees (colors: green = left hemisphere; violet = right hemisphere). The first dimension also shows significant interspecies differences between humans and chimpanzees (colors: yellow = chimpanzees; blue = humans). Anatomical and morphological feature labels have been added at the center of each axis to facilitate interpretation of the embedding dimensions.

In the case of the frontal aslant tracts (FAT), the bundles in both species visually appear quite similar, extending to two regions of the frontal cortex. However, analysis of the first dimension reveals intriguing morphological differences between them (see Fig. 11).

Indeed, this dimension uncovers subtle variations in the morphology of the FATs that may not be immediately apparent from visual inspection alone. While the general trajectory and connectivity of the tracts are conserved, the detailed shape and spatial characteristics of the bundles exhibit species-specific features.

The analysis shows that the FATs of both species display a range of shapes along the principal axis. As one progresses along this axis, the bundles transition from a flat, “open” shape to a more curved morphology. Notably, chimpanzees exhibit considerable variability in the morphological profile of their FATs, with some bundles appearing very flat while others are distinctly curved. In contrast, human subjects show less variability, with most FATs concentrated toward the left side of the axis and exhibiting a relatively flat shape (independent t-test: t(76) = 16.0, p = $1.2\times 10^{-33}$ , see Fig. 11, second row). This observation is supported by the significant lateralization pattern of the FATs in chimpanzees (paired t-test: t(38) = -2.1, p = $4.1\times 10^{-2}$ , see Fig. 11, first row), as seen in dimension 6 of the isomap. Specifically, bundles in the left hemisphere tend to display more closed and twisted morphologies than those in the right hemisphere.

#### 3.3.3 The uncinate fasciculus (UF)

The uncinate fasciculus (UF) comprises curved white matter bundles that connect the rostral part of the temporal lobes. These bundles curve upward from the anterior insula and extend to the inferior frontal gyri and the orbitofrontal cortex. Traditionally, the UF is considered part of the limbic system and is thought to play a critical role in integrating mnemonic representations from the temporal lobe with decision-making processes in the frontal lobe (Von Der Heide et al., 2013). Its connections between the temporal and frontal regions facilitate the interaction between memory and decision making, which is essential for complex behaviors and social interactions. Disruptions or alterations in the UF have been associated with neurodevelopmental and psychiatric disorders, underscoring its significance in both normal cognitive function and pathological conditions (Olson et al., 2015).

**Fig. 12.**
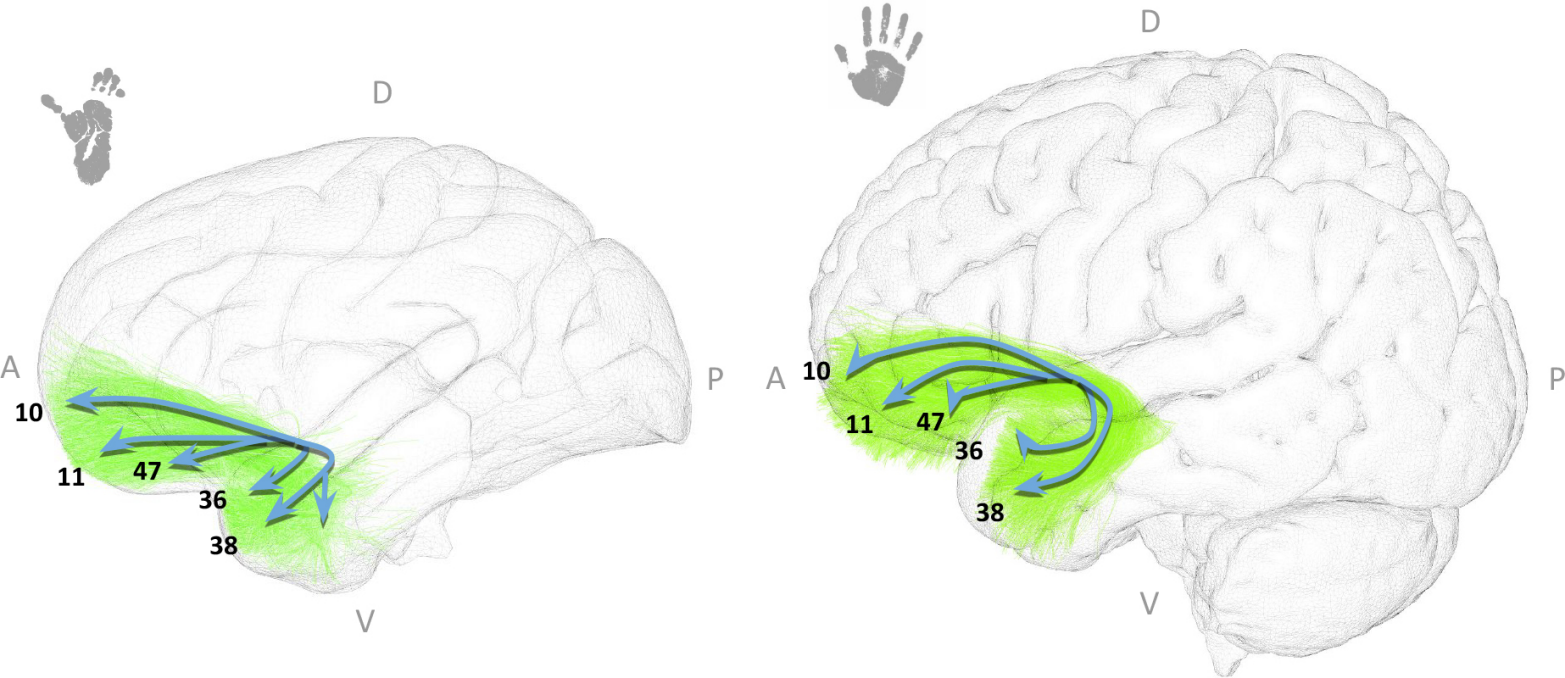
The uncinate fasciculus in chimpanzees (left) and humans (right). Both species’ bundles are displayed in their respective templates (Juna.Chimp for chimpanzees and MNI for humans). Anatomical orientation labels are indicated as A (anterior), P (posterior), D (dorsal), and V (ventral). Numbers correspond to the Brodmann areas reached by the bundle extremities. Blue arrows are schematic visual aids highlighting the main bundle trajectories and cortical targets reached by each bundle extremity.

In our study, the UF in both species connects similar regions, with fibers originating in BAs 36 and 38 of the temporal lobe, crossing the insula, and reaching BAs 10, 11, and 47 of the middle and inferior frontal cortex (see Fig. 12).

##### 3.3.3.1 Comparative morphology using isomap

**Fig. 13.**
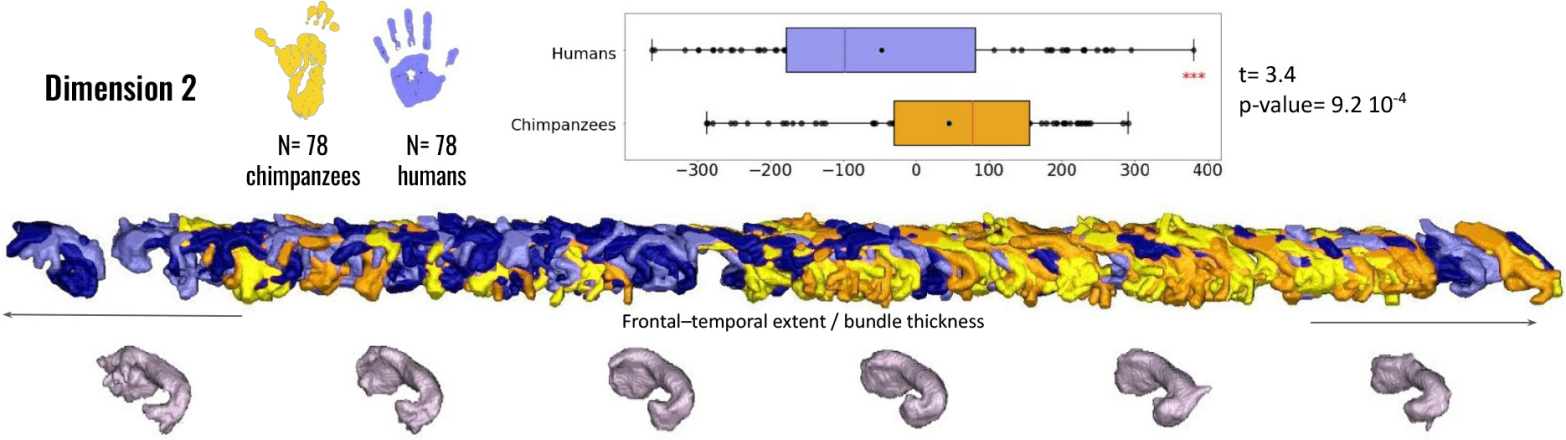
Geometrical analysis of the uncinate fasciculus (UF) between species. The second dimension of the isomap reveals significant interspecies differences between humans and chimpanzees (colors: yellow = chimpanzees; blue = humans). Anatomical and morphological feature labels have been added at the center of each axis to facilitate interpretation of the embedding dimensions.

No inter-hemispheric differences were observed within either the human or chimpanzee species. However, when considering both species together, along the axis of the second dimension of the embedding, the uncinate fasciculus in chimpanzees tends to reach fewer regions of the inferior frontal cortex than in humans. In humans, the bundles appear thicker and spread more extensively into both the temporal and frontal cortices (independent t-test: t(76) = 3.4, p = $9.2\times 10^{-4}$ , see Fig. 13).

#### 3.3.4 The inferior fronto-occipital fasciculus (IFOF)

The inferior fronto-occipital fasciculus (IFOF) is a long and thick association fiber tract that connects the occipital lobe and the frontal lobe passing through the temporal lobe. This tract runs deep to the insula and traverses the extreme and external capsules (Martino et al., 2010). In humans, the IFOF is involved in various cognitive functions, particularly the integration of visual information with higher-order cognitive processes. It also plays a crucial role in language processing, visual perception, and visuo-spatial attention (Andelman-Gur et al., 2020; Herbet, Moritz-Gasser, et al., 2017; Herbet, Yordanova, et al., 2017).

**Fig. 14.**
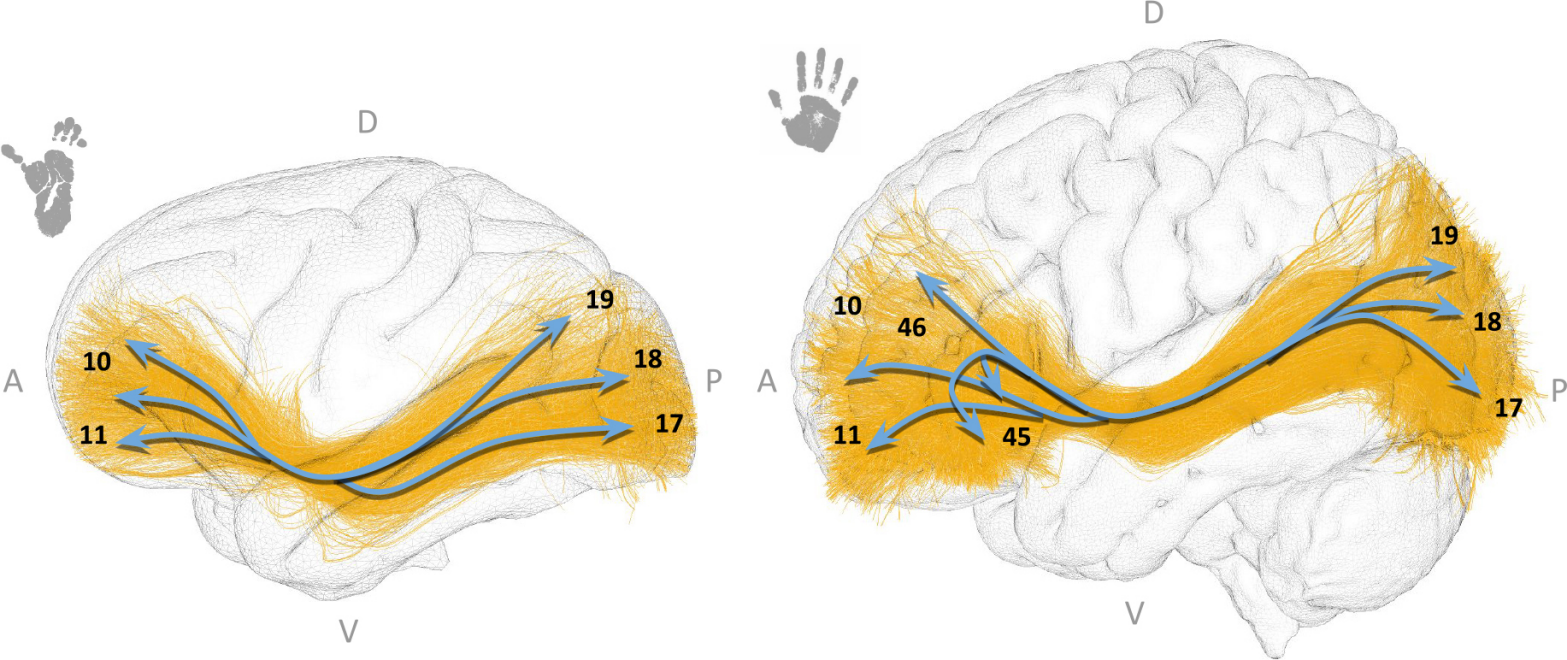
The inferior fronto-occipital fasciculus (IFOF) in chimpanzees (left) and humans (right). Both species’ bundles are displayed in their respective templates (Juna.Chimp for chimpanzees and MNI for humans). Anatomical orientation labels are indicated as A (anterior), P (posterior), D (dorsal), and V (ventral). Numbers correspond to the Brodmann areas reached by the bundle extremities. Blue arrows are schematic visual aids highlighting the main bundle trajectories and cortical targets reached by each bundle extremity.

In this study, the IFOF in chimpanzees and humans appear very similar in their trajectory, with a more widespread projection within the frontal lobe in humans. They connect the occipital lobe (BAs 17, 18, and 19 in both species) to the frontal regions to reach BAs 10 and 11 in chimpanzees, and BAs 10, 11, 45, and 46 in humans (see Fig. 14).

##### 3.3.4.1 Comparative morphology using isomap

**Fig. 15.**
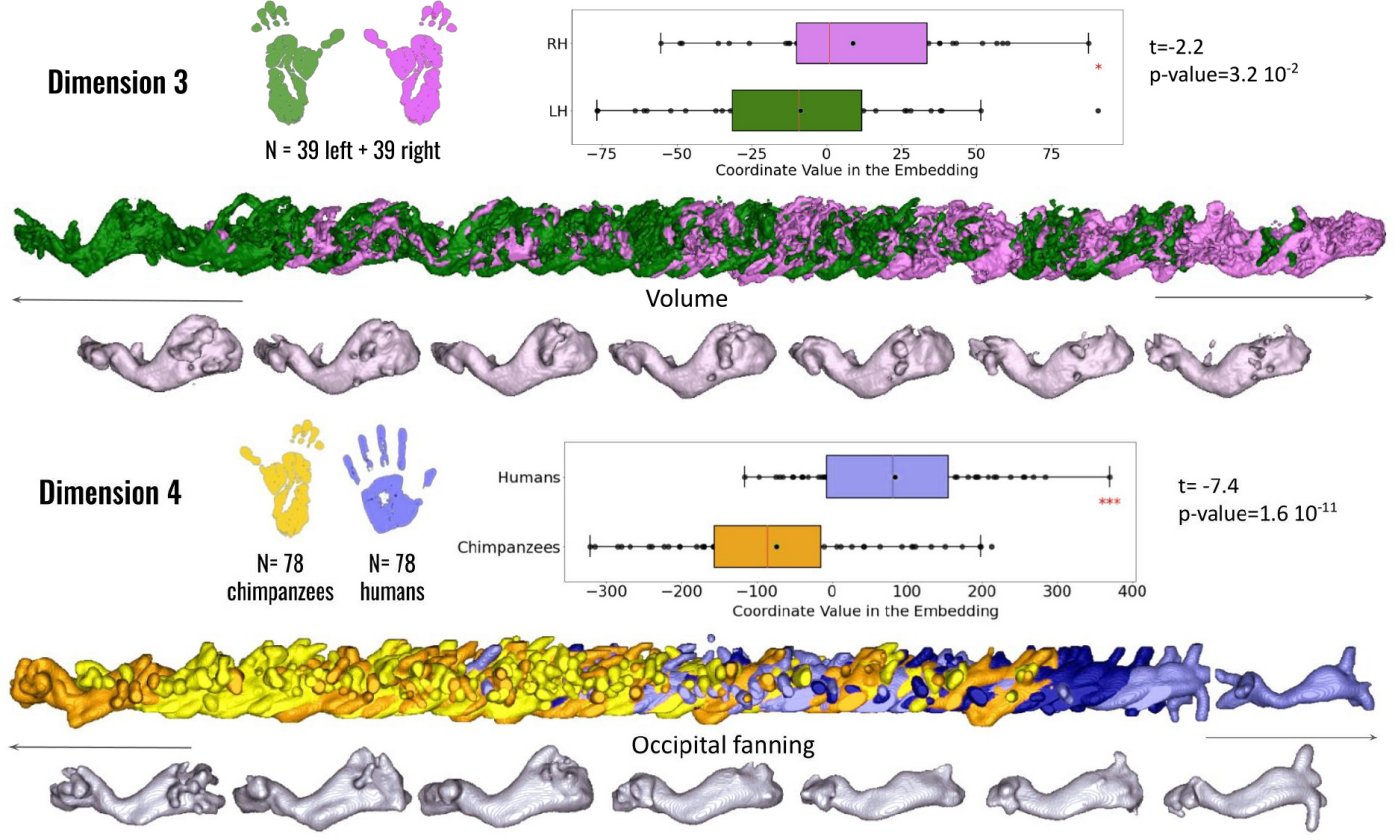
Geometrical analysis of the inferior fronto-occipital fasciculus (IFOF) between species. The third dimension of the isomap reveals significant hemispheric differences within chimpanzees (colors: green = left hemisphere; violet = right hemisphere). The fourth dimension reveals significant interspecies differences between humans and chimpanzees (colors: yellow = chimpanzees; blue = humans). Anatomical and morphological feature labels have been added at the center of each axis to facilitate interpretation of the embedding dimensions.

The isomap algorithm revealed differences between the left and right hemisphere bundles in chimpanzees (but none in humans) (paired t-test: t(38) = 2.2, p = $3.2\times 10^{-2}$ , see Fig. 15, first row). Specifically, the left IFOF appears thicker and has a greater volume than the right IFOF, attributed to its extended occipital connections.

When comparing both species together, the chimpanzee IFOF seems to exhibit a greater fanning of fibers at the occipital end than the human IFOF (independent t-test: t(76) = -7.4, p = $1.6\times 10^{-11}$ , see Fig. 15, second row).

## 4 Discussion

This study investigated the morphological features of deep white matter bundles (DWMBs) in humans and chimpanzees using diffusion MRI, with a focus on identifying structural differences that may reflect evolutionary adaptations in brain connectivity. Leveraging species-specific atlases, we delineated and analyzed frontal DWMBs implicated in higher-order cognition, both within and across species.

Among the four studied bundles, the arcuate fasciculus showed the most pronounced inter-species differences in shape and trajectory. In humans, the AF is significantly larger and includes a marked ventral component that links the caudal temporal cortex with the inferior frontal lobe. In chimpanzees, the AF lacks a clear ventral pathway and follows a more restricted dorsal route, corroborating previous findings in the two species (Bryant et al., 2020; Rilling et al., 2007, 2008). While the AF is not directly responsible for speech production, which relies on cortico-subcortical pathways including connections between Broca’s area and brainstem nuclei, it plays a central role in language processing, including syntactic integration and phonological working memory. Thus, species differences in AF structure may support the evolution of complex language functions in humans, even if it is not sufficient to explain the lack of speech in chimpanzees.

Our isomap-based morphometric analysis effectively distinguished species based on AF shape features such as volume and curvature. Within humans, we found strong leftward asymmetry in the AF, reinforcing its association with language lateralization (Geschwind, 1970; Ivanova et al., 2021). This asymmetry was not found in chimpanzees, in line with the lack of hemispheric specialization for language and their reliance on gestural and non-verbal vocal communication (Lyn, 2012). Furthermore, previous studies have shown that connected regions of the arcuate fasciculus, such as BA44 and BA45 (Broca areas), were significantly larger in humans than in chimpanzees and that humans develop BA44 and BA45 asymmetries while chimpanzees do not (Schenker et al., 2010). Thus, the observed absence of lateralization of the chimpanzee AF could be directly linked to the absence of asymmetry in the extension of their connections to homologous cortical areas between the two hemispheres observed with our cohort. Importantly, our analysis of the arcuate fasciculus replicated previously reported species differences in size, trajectory, and lateralization (Bryant et al., 2020; Rilling et al., 2008, 2012), providing an internal validation of our methodological approach. The fact that the isomap-based framework successfully recovered these well-established anatomical distinctions supports its reliability in capturing meaningful inter-species variation. This, in turn, increases confidence in the interpretability of the findings for the other, less well-characterized bundles analyzed in this study. Nevertheless, it is important to acknowledge that the morphology of the arcuate fasciculus in chimpanzees remains a debated topic in the literature. Differences between our reconstruction and the ones described in Roumazeilles et al. (2020), for example, likely reflect both methodological and biological factors. While Roumazeilles et al. presented ROI-based segmentation, our approach in constructing our atlases is based on fiber clustering, using a species-specific template (see Chauvel et al. (2023) for details), which may lead to distinct tract delineations. This highlights how sensitive the AF (or other bundles) is to tractography protocols and resolution constraints in chimpanzees, emphasizing the need for harmonized reconstruction strategies and systematic cross-study comparisons.

The frontal aslant tract (FAT) remains a relatively new and debated fiber pathway, first described in humans in 2007 (Aron et al., 2007), and subsequently identified in macaques (Bryant et al., 2020; de Schotten et al., 2012). The observed pathways suggest roles in motor control and coordination similar to those in humans. However, the precise cognitive and motor functions associated with the FAT in primates are not well defined (Barrett et al., 2020). The isomap analysis revealed significant differences between the FATs of chimpanzees and humans. While both species exhibit connections between the inferior and superior frontal gyri, the human FAT extends further into superior frontal areas, particularly in the regions of the pre-Supplementary Motor Area (pre-SMA) and Supplementary Motor Area (SMA). This finding is consistent with earlier studies in macaques, where connections of the FATs to the pre-SMAs were found to be thinner and less consistent (Schmahmann et al., 2009). This increased connectivity in humans may also correlate with enhanced executive functions, speech, and social behaviors observed, which are less pronounced in chimpanzees. Interestingly, chimpanzees displayed greater FAT shape variability, especially on the left hemisphere, whereas human FATs showed more consistent patterns across individuals. Indeed, along the first isomap dimension, human FATs cluster more tightly than chimpanzee FATs, indicating less inter-individual morphological variability along this axis. This could indicate a more stable frontal connectivity profile in humans and potentially reflects species-specific differences in sulcal organization, which remains poorly explored in chimpanzees.

The uncinate fasciculus is well defined in humans as a white matter tract connecting the frontal and temporal lobes. It is implicated in various psychiatric and developmental disorders, and damage to this structure is often associated with deficits in memory, language, and socio-emotional processing. Although the cognitive and emotional roles of the UF in non-human primates are inferred from anatomical studies, they are not as thoroughly characterized as in humans (Von Der Heide et al., 2013). In both species, the UF links similar regions, consistent with previous research (Bryant et al., 2020; Hecht et al., 2015). However, humans exhibit a thicker UF with more extensive connections, particularly within the temporal and frontal cortices. This finding suggests that the human UF may support more complex mnemonic and decision-making processes than chimpanzees, which are essential for advanced social interactions and cognitive functions (Aron et al., 2007; Olson et al., 2015; Schmahmann et al., 2009).

The inferior fronto-occipital fasciculus, in humans, connects the frontal cortex with the occipital cortex and plays a significant role in integrating visual information with cognitive processes such as verbal semantic elaboration and language processing (Sarubbo et al., 2013). The presence and functional significance of the IFOF remain a point of debate in non-human primates. Some studies suggest that similar tracts in non-human primates may not be as prominent or may function differently, raising questions about the evolutionary development and specific roles of these pathways across species. This ongoing debate underlines the complexity of comparing neural structures and their functions between humans and other primates without an adequate analysis framework.

In Old-World monkeys, the IFOF is believed to support basic communication and visual processing, with ventral pathways being utilized for these functions (Schoenemann, 2012). However, the exact role of the IFOF-like pathway in semantic communication and its functional equivalence to the human IFOF remains unclear. Our results show that chimpanzee IFOFs are thicker with stronger occipital connectivity, particularly in the left hemisphere. In contrast, the human IFOF showed no significant lateralization, potentially reflecting species-specific adaptations in visual processing and visuo-spatial attention. These findings suggest that, while the IFOF serves similar fundamental functions across species, structural adaptations in chimpanzees may enhance complex visual and cognitive integration. One might speculate that in natural environments, chimpanzees rely heavily on visuo-spatial attention (Dolins et al., 2014; Rosati & Hare, 2012) for activities such as locating food and avoiding predators, which could explain these structural differences.

### 4.1 Limitations

While this study provides valuable insights into the morphological characteristics of DWMB in humans and chimpanzees, several limitations should be acknowledged.

The Ginkgo Chauvel human and chimpanzee atlases used in this study, while established using the same approach, may not capture all individual variations within each species. The atlases were designed based on specific criteria, and their ability to generalize findings to broader populations is inherently limited. In addition, the lower resolution of diffusion MRI data in chimpanzee subjects may affect the accuracy of fiber reconstruction at the cortical ribbon level.

While the UF has been described in previous studies to comprise medial and lateral segments with distinct connectivity patterns (Baydin et al., 2025; Hau et al., 2017), our analysis treated the UF as a single bundle due to limitations in reliably segmenting these subcomponents across all subjects. Consequently, the observed differences in human versus chimpanzee UF morphology should be interpreted as reflecting the overall bundle rather than segment-specific effects.

One of the key limitations of this study is the lack of functional data to support the morphological conclusions drawn. In this study, while structural differences in white matter bundles have been highlighted, the functional implications of these differences remain speculative. In future research, incorporating behavioral data to compensate for the lack of functional MRI data could provide a more holistic understanding of how these structural variations in DWMBs translate to functional disparities between species.

The registration process, despite its rigor, presents challenges, especially when aligning the brains of two distinct species. Although the sulci-based registration techniques used were innovative, some inaccuracies, particularly in the anterior inferior frontal cortex, were observed. These inaccuracies may affect the precise localization and comparison of white matter bundles. To mitigate this, we applied additional alignment steps, including PCA-based shape registration, to improve correspondence across species. While localized differences should be interpreted with caution, the overall morphological patterns and cross-species trends identified in this study remain robust. The combination of high-dimensional isomap embeddings and multiple alignment strategies ensures that the main conclusions regarding bundle morphology and interspecies differences are preserved.

It should be noted that the chimpanzee scans were acquired under anesthesia, whereas human participants were awake. Although anesthesia may influence functional measurements, its effect on the diffusion-based structural analyses conducted here is expected to be minimal. However, we cannot completely rule out subtle influences on white matter microstructure or tractography results.

Last, the study was conducted on a limited number of individuals from both species. This small sample size may not fully represent the diversity within human and chimpanzee populations. Future research should include a larger sample to validate the findings and ensure they are representative of the species as a whole. Additionally, considering age, sex, and developmental stages could provide deeper insights into the variability and evolution of white matter structures, and would allow to separate evolutionary traits from developmental traits.

### 4.2 Perspectives

Beyond the global morphometric framework presented here, future work could integrate complementary tractometry approaches such as BUAN (Chandio et al., 2020), which allow for segment-wise comparisons of streamline profiles along the tract trajectory. While such methods are particularly informative within a single species, their application to cross-species datasets remains challenging due to the lack of guaranteed pointwise homology along the bundles. Nevertheless, combining our surface-based embedding approach with segment-level analytic frameworks could, in future studies, provide a multi-scale understanding of both global and local morphological differences across species.

The observed morphological differences in these white matter bundles provide insights into the neural substrates underlying species-specific cognitive and behavioral functions. The more complex and extensive white matter tracts in humans likely support advanced language, cognitive functions, and social behaviors, which are less pronounced or absent in chimpanzees.

Future research should focus on elucidating the functional implications of these structural variations by incorporating behavioral data. Expanding studies to include larger and more diverse samples, as well as examining a wider array of primate species, will provide a more comprehensive understanding of the evolutionary development of these neural pathways. Investigating the genetic and developmental factors that drive these structural differences will also be crucial in uncovering the neuro-biological foundations of human cognition and behavior.

## 5 Conclusion

Overall, our findings support our hypotheses that specific frontal white matter bundles exhibit marked differences in structure and asymmetry between humans and chimpanzees. The expansion and leftward lateralization of the AF, the morphological consistency of the FAT in humans, and the increased volume and connectivity of the UF align with the proposed link between white matter architecture and species-specific cognitive abilities. Observed differences in the IFOF, including its lateralization in chimpanzees, suggest visual processing adaptations that may reflect distinct ecological demands.

## Supplementary Material

Supplementary Material

## Data Availability

The chimpanzee DWMB atlas used in this study is available on the Zenodo platform at https://doi.org/10.5281/zenodo.7147789. The human DWMB is available on the Zenodo platform at https://doi.org/10.5281/zenodo.7308510. The results of the registration between human and chimpanzee as well as transformation files are available on zenodo at https://doi.org/10.5281/zenodo.13935442. The construction of atlases is based on the analysis of the anatomical and diffusion MRI dataset using the tractography and fiber clustering tools available from the Ginkgo toolbox (Ginkgo Team, BAOBAB, NeuroSpin, Paris-Saclay University, CNRS, CEA, https://framagit.org/cpoupon/gkg). The isomap algorithm used for this analysis is given on the neurospin/github repository: https://github.com/neurospin/point-cloud-pattern-mining.
