# Supplementary material for: Evolutionary signatures in deep white matter architecture: A comparative study of humans and chimpanzees
Source: Imaging Neurosci (Camb). 2026 Mar 11;4:IMAG.a.1154. doi: 10.1162/IMAG.a.1154 (PMC12980544; doi:10.1162/IMAG.a.1154)
Supplement: Supplementary Material [file IMAG.a.1154_supp.pdf]

Supplementary material

SM 1 - Table of distances between the atlases' bundles and the individual tractograms used for the fiber labeling step

The cross-subject variability in the shape and position of the bundles must be taken into account. The level of variability also depends on the bundle type, with some being more variable in shape than others. To address this, a threshold distance in millimeters was empirically chosen and further optimized for each white matter bundle. This was determined from a series of labeling experiments with a subset of individuals to establish the optimal threshold distance for each white matter bundle. The set of distances was stored in a file provided as input to the algorithm (see Supplementary materials, Figure S1).

	Distances (mm)	
**Deep white matter bundle** (for left and right hemispheres)	**Chimpanzee**	**Human**
Anterior commissure	5	10
Anterior thalamic radiations	8	10
Arcuate	4.5	12
Corpus callosum (all components)	4	8
Cortico-spinal	7	5
Dorsal cingulum	5	7
Fornix	5.5	8
Frontal aslants	6.5	7
Inferior fronto-occipital	8	7
Inferior longitudinal	3.5	10
Middle longitudinal	5.5	13
Optical radiations	3.5	10
Posterior thalamic radiations	5	10
Superior thalamic radiations	5	10
Uncinate	4	12
Ventral cingulum	6.5	13
Ventral visual stream	6	10

Figure S1: Table of distances for the application of the deep white matter bundles atlases on the chimpanzee and human subjects.

SM 2 - Validation of the atlases application on both species

Both the chimpanzee and human atlases were applied to the 39 chimpanzee and the 39 human subjects respectively. All bundles from the atlas were recovered for all subjects, see example on Figures S2 and S3.

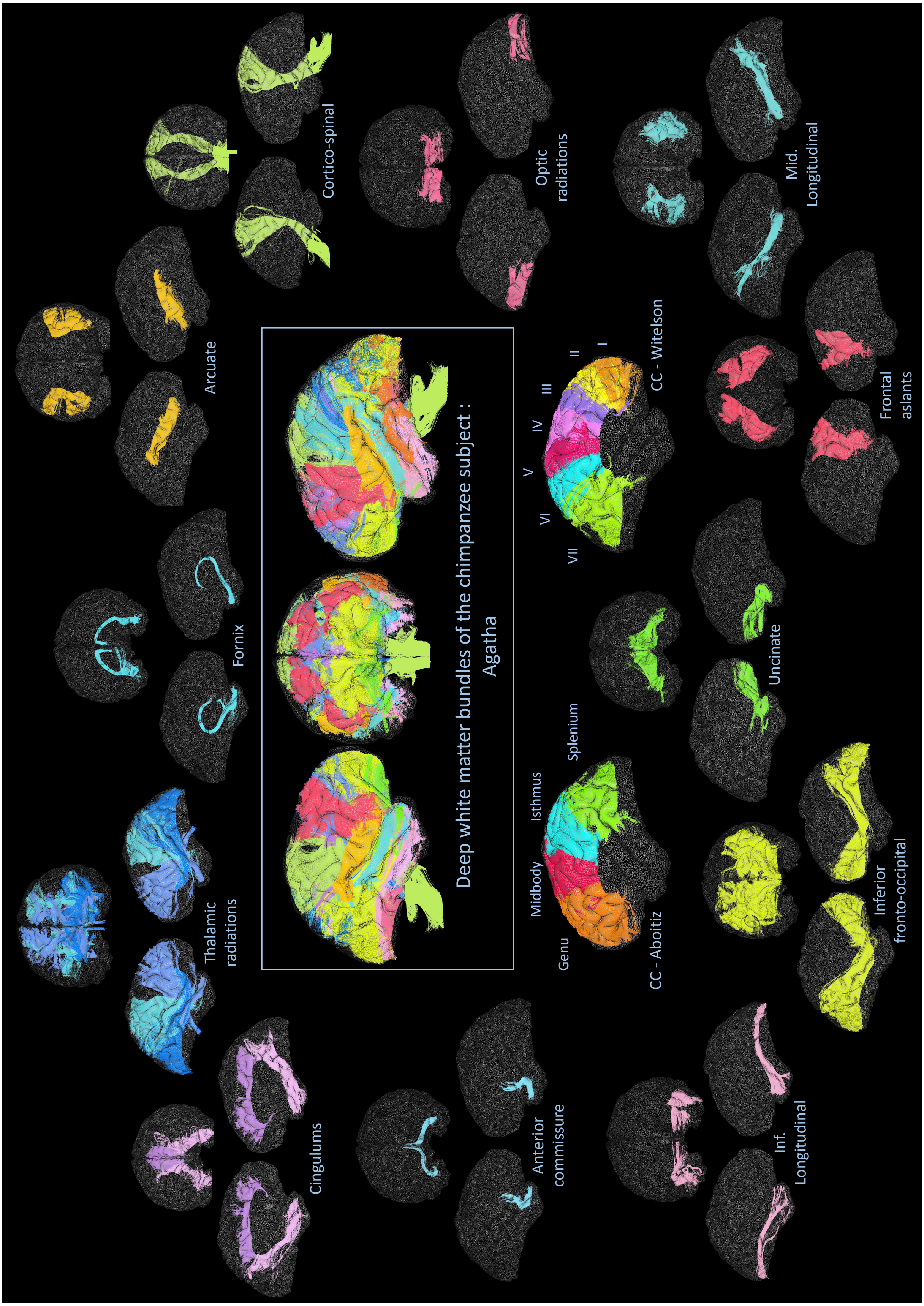

Figure S2: ***Deep white matter bundles of the chimpanzee called "Agatha".*** *Example of the application of the deep white matter chimpanzee atlas using the described algorithm and the distance file provided the result depicted in this figure for the chimpanzee subject : Agatha.*

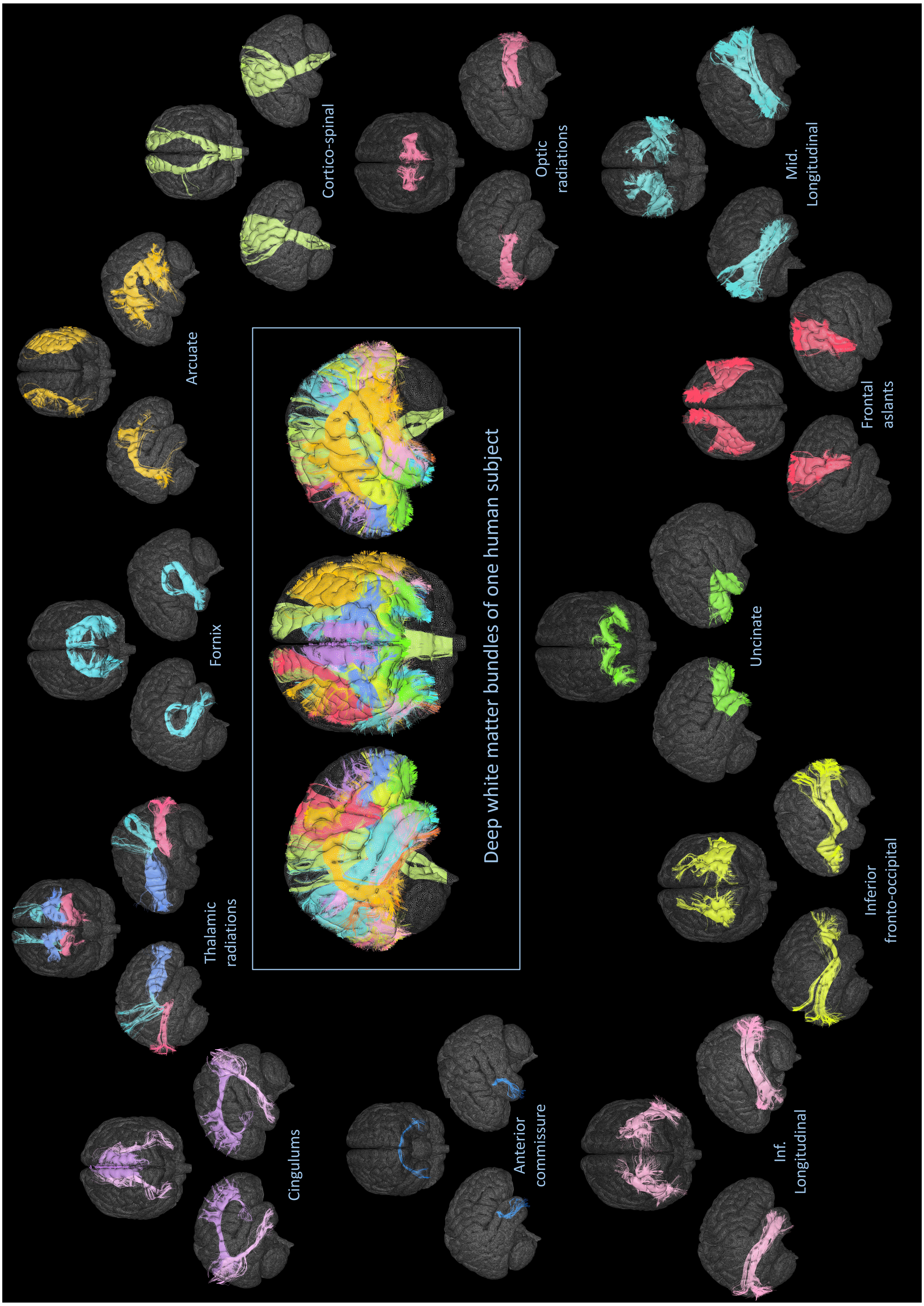

Figure S3: ***Deep white matter bundles of one human subject (subject 100408)****. Example of the application of the deep white matter human atlas using the described algorithm and the distance file provided the result depicted in this figure for the human subject 100408 of the HCP database.*

SM-3: Validation of the human deep white matter atlas' tracts

To provide additional confidence in the anatomical accuracy of the human DWMB atlas used in this study, we compared the major tracts included in our atlas with those from established human tractography atlases (Guevara et al., 2012; Herlin et al., 2024) and available in the same template space (the MNI ICBM 2009c non-linear asymmetric template). Specifically, we focused on the arcuate fasciculus (AF), uncinate fasciculus (UF), and inferior fronto-occipital fasciculus (IFOF). Each tract is presented in a separate subsection with a figure showing an overlay between our atlas and the ones from Guevara et al., 2012 and Herlin et al., 2024 when possible.

SM-3.1 Arcuate Fasciculus (AF)

The human arcuate fasciculus (AF) in our DWMB atlas connects the inferior frontal gyrus and ventral premotor cortex with posterior and inferior regions of the temporal lobe, arching around the Sylvian fissure. In this work, we focus on the main section of the bundle and do not separate its potential subcomponents. Figure S4 shows an overlay of our AF (orange) and the AF from the Guevara et al. atlas and Herlin et al. (blue) in the MNI space. Frontal and temporal terminations, as well as the overall curvature, are highly consistent between the atlases, supporting the anatomical validity of our AF delineation. The fiber's trajectory of the AF closely follows previous descriptions in the literature (Bernard et al., 2019; Catani & De Schotten, 2008).

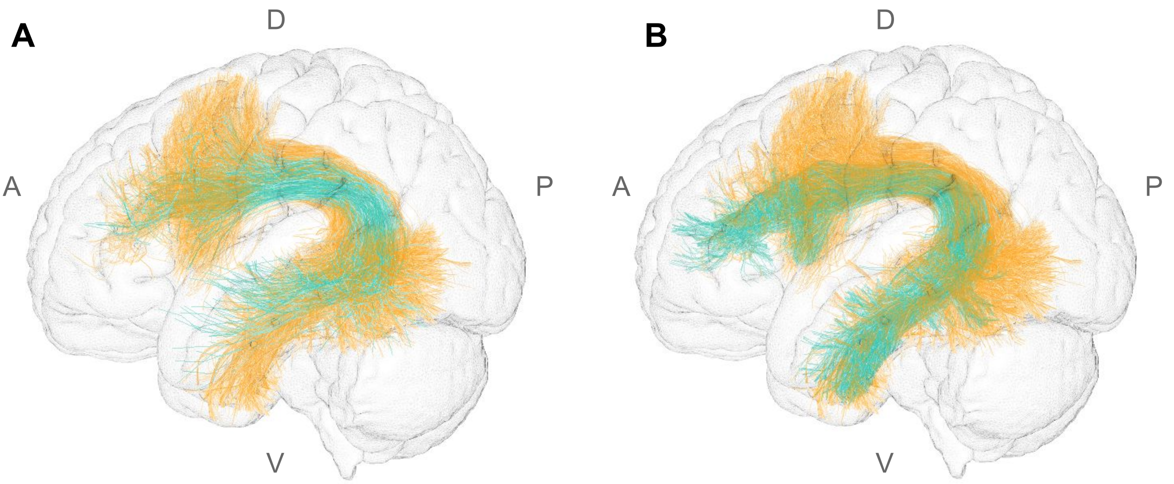

Figure S4: ***Validation of the human arcuate fasciculus (AF) in the DWMB atlas.*** *Sagittal view showing the AF from our human DWMB atlas (orange) overlaid with the corresponding tract from Guevara et al., 2012(A, blue) and Herlin et al., 2024(B; blue), projected onto the cortical mesh of the MNI template. Anatomical orientation labels are indicated as A (anterior), P (posterior), D (dorsal), and V (ventral). This comparison demonstrates the spatial correspondence of the AF trajectories and terminations between the different atlases, supporting the anatomical validity of the DWMB delineation.*

SM-3.2 Uncinate Fasciculus (UF)

Figure S5 presents the UF from our atlas (orange) overlaid with the UF from Guevara et al. (blue) and Herlin et al. (blue). The course of the UF connecting the anterior temporal lobe to the orbito-frontal cortex is well-matched between the different atlases. Minor differences in lateral spread are observed, likely reflecting differences in tractography parameters and streamline clustering approaches. The observed trajectory also matches the one described in Catani and De Schotten, 2008.

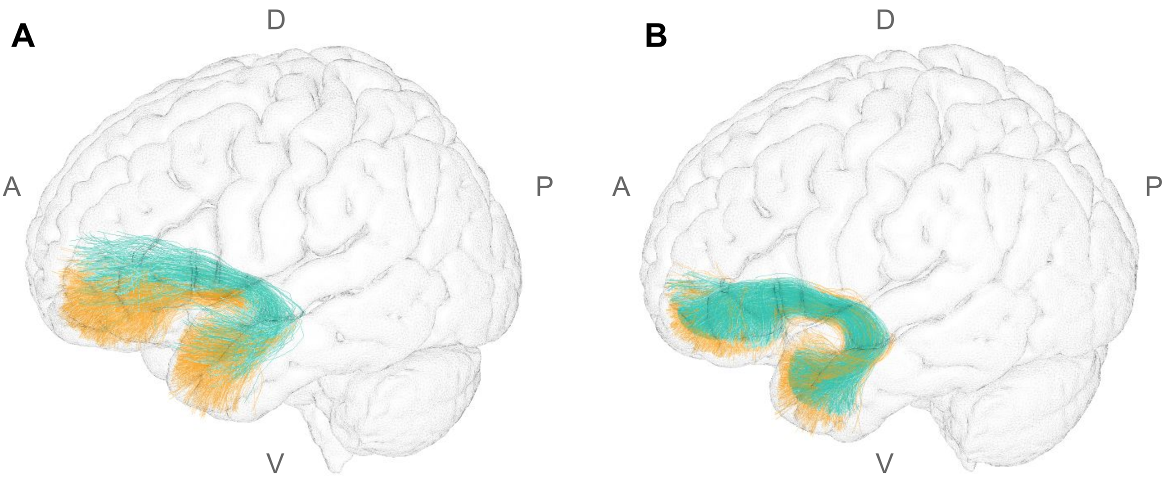

Figure S5: ***Validation of the human uncinate fasciculus (UF) in the DWMB atlas.*** *The figure shows the UF from our human DWMB atlas (orange) overlaid with the corresponding tract from Guevara et al., 2012(A, blue) and Herlin et al., 2024(B; blue), projected onto the cortical mesh of the MNI template in a sagittal view. Anatomical orientation labels are provided as A (anterior), P (posterior), D (dorsal), and V (ventral). This comparison demonstrates the spatial correspondence of the UF trajectories and terminations between the different atlases, supporting the anatomical validity of the DWMB delineation.*

SM-3.3 Inferior Fronto-Occipital Fasciculus (IFOF)

The IFOF from our DWMB atlas shows strong agreement with the IFOF from the Guevara et al., 2012 and the Herlin et al., 2024 atlases (Figure S6). All atlases depict a ventral route connecting the frontal and occipital lobes, with similar anterior-posterior extent and curvature. Small discrepancies in dorsal terminations are observed and likely reflect individual variability or methodological differences in tract reconstruction.

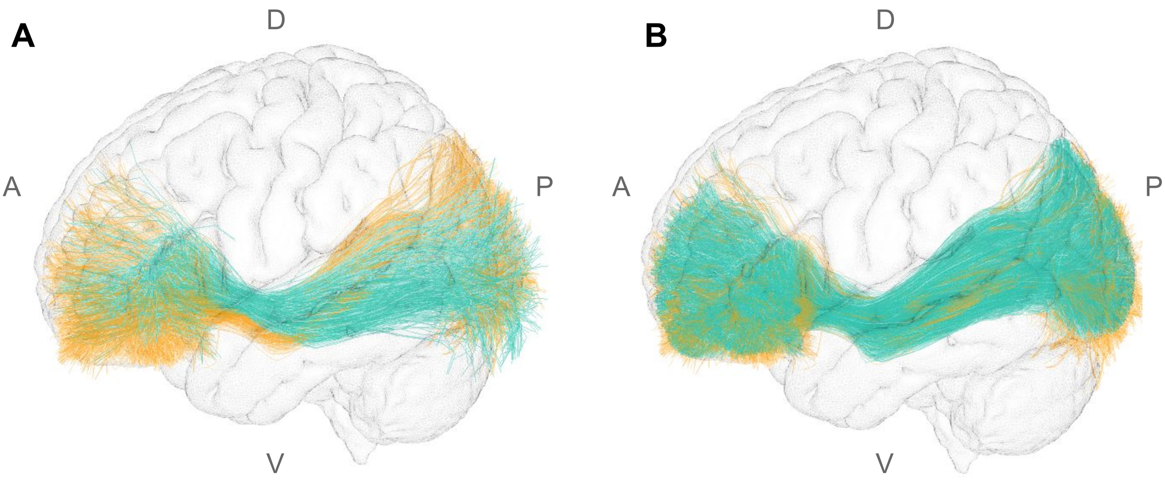

Figure S6: ***Validation of the human inferior fronto-occipital fasciculus (IFOF) in the DWMB atlas.*** *The figure shows the IFOF from our human DWMB atlas (orange) overlaid with the corresponding tract from Guevara et al., 2012(A, blue) and Herlin et al., 2024(B; blue), projected onto the cortical mesh of the MNI template in a sagittal view. Anatomical orientation labels are provided as A (anterior), P (posterior), D (dorsal), and V (ventral). This comparison demonstrates the spatial correspondence of the IFOF trajectories and terminations between the different atlases, supporting the anatomical validity of the DWMB delineation.*

SM-3.4 Frontal Aslant Tract (FAT)

The frontal aslant tract (FAT) is not included in the Guevara et al. atlas. Nevertheless, the tract pathway is consistent with previous descriptions from the literature (Briggs et al., 2020; La Corte et al., 2021) and with the atlas from Herlin et al., 2024. Indeed, the FAT presented in our atlas connects the superior frontal cortex (pre-SMA and SMA) to the inferior frontal gyrus (see Figure S7). The FAT delineation in our atlas reproduces the expected connected regions, trajectory, and curvature, providing confidence in its anatomical validity.

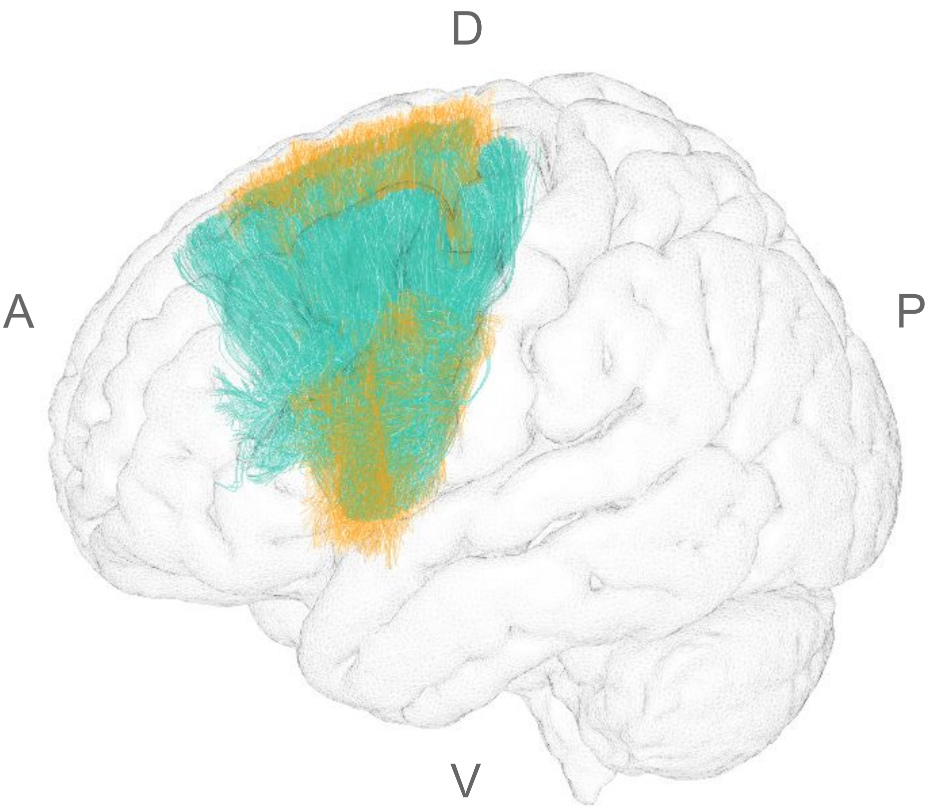

Figure S7: ***Representation of the human frontal aslant tract (FAT) in the DWMB atlas.*** *The FAT is shown in orange, overlaid with the corresponding tract from 2024 in blue, projected onto the cortical mesh of the MNI template in a sagittal view. Anatomical orientation labels are provided as A (anterior), P (posterior), D (dorsal), and V (ventral).*

SM 4 - Assessment of Cross-Species Registration Accuracy

To evaluate the quality of our cross-species registration, we visualized the alignment of the human and chimpanzee cortical surfaces and whole-brain meshes. While the sulci-based registration approach generally ensured good correspondence between homologous regions, localized misalignments were observed, particularly in the anterior inferior frontal cortex. These discrepancies are expected due to the substantial expansion of the human frontal cortex relative to chimpanzees and are important to consider when interpreting bundle trajectories terminating or originating in this region. The figure below illustrates the registration quality across multiple views and levels of detail. Top panels show three orthogonal planes (axial, coronal, sagittal) of the average cortical ribbon for each species after registration (chimpanzee in pink, human in gray). Middle panels display the full brain meshes overlaid for the same planes. Bottom panels

provide an enlarged view of the overlaid brain meshes, with yellow arrows highlighting regions where the registration shows notable deviations. Anatomical orientation labels (A = anterior, P = posterior, D = dorsal, V = ventral) have been added for reference. These visualizations allow readers to assess spatial alignment and understand potential limitations in areas most affected by interspecies anatomical differences.

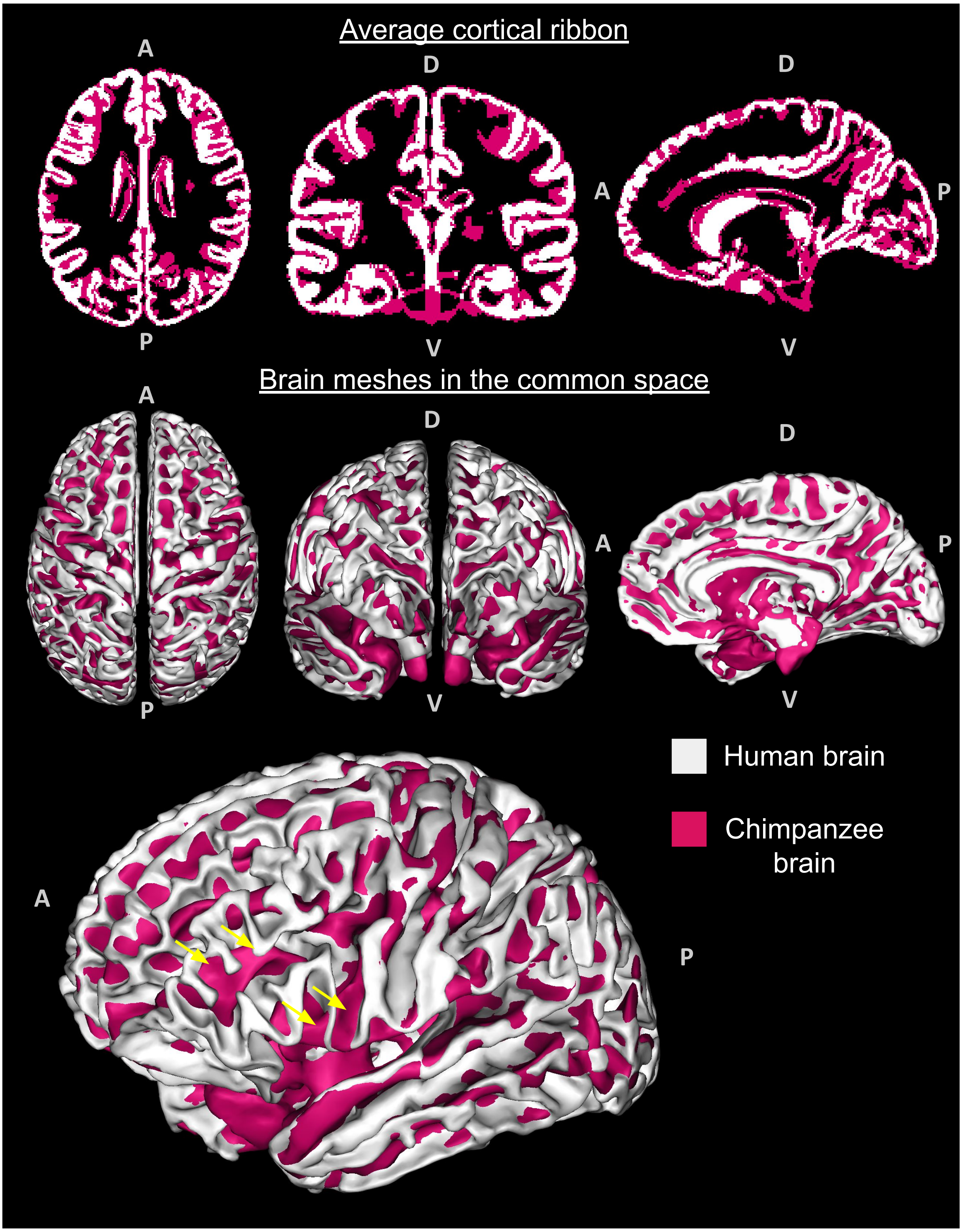

Figure S8: ***Assessment of cross-species registration accuracy.*** *Top row: three orthogonal planes (axial, coronal, sagittal) of the average cortical ribbon for chimpanzee (pink) and human (gray) after registration. Middle row: overlaid full brain meshes in the same planes, showing general alignment. Bottom: enlarged overlay of brain meshes with yellow arrows indicating regions of imperfect registration, particularly in the anterior inferior frontal cortex. Anatomical orientation labels (A = anterior, P = posterior, D = dorsal, V = ventral) have been added at the center of each axis to facilitate interpretation. These visualizations illustrate the spatial accuracy of the registration and highlight areas where anatomical differences between species may affect bundle alignment.*

SM-5: False Discovery Rate (FDR) Correction for Multiple Comparisons

To limit the risk of false positives arising from multiple statistical tests performed across bundles and Isomap dimensions, we applied a False Discovery Rate (FDR) correction. The procedure of Benjamini and Hochberg (1995) was chosen because it controls the expected proportion of false discoveries among all significant results, while maintaining greater sensitivity than conservative approaches such as Bonferroni correction. This method is well suited for our analyses, which involve interrelated morphological measures.

Raw p-values were ranked in ascending order and each compared to a critical value $(i/m) \times 0.05$, where i is the rank and m the total number of tests. The largest p_i satisfying $p_i \leq (i/m) \times 0.05$ and all smaller p-values were considered significant. Adjusted p-values were computed as $p_{\text{adj}}(i) = \min_{j \geq i}(m/j) \times p_j$, ensuring monotonicity across ranks.

Test	Raw p	BH adjusted p
AF human dimension 1	4.4×10^{-3}	5.9×10^{-3}
AF human dimension 2	3.4×10^{-3}	5.4×10^{-3}
AF between species	4.1×10^{-39}	3.28×10^{-38}
FAT chimp dimension 6	4.1×10^{-2}	4.1×10^{-2}
FAT between species	1.2×10^{-33}	4.8×10^{-33}
UF between species	9.2×10^{-4}	1.84×10^{-3}
IFOF chimp dimension 3	3.2×10^{-2}	3.66×10^{-2}
IFOF between species	1.6×10^{-11}	4.27×10^{-11}

Table S1: Summary of statistical tests on DWMBs with FDR correction.
Raw p-values and Benjamini-Hochberg (BH) FDR-adjusted p-values are reported for each comparison. Tests include within-species hemispheric differences and between-species differences. All reported BH-adjusted p-values correspond to a false discovery rate threshold of $p = 0.05$.

References

Bernard, F., Zemmoura, I., Ter Minassian, A., Lemée, J.-M., & Menei, P. (2019). Anatomical variability of the arcuate fasciculus: A systematical review. *Surgical and radiologic anatomy*, *41*, 889–900.

Briggs, R. G., Khan, A. B., Chakraborty, A. R., Abraham, C. J., Anderson, C. D., Karas, P. J., Bonney, P. A., Palejwala, A. H., Conner, A. K., O'Donoghue, D. L., & Sughrue, M. E. (2020). Anatomy and white matter connections of the superior frontal gyrus. *Clinical Anatomy*, *33*(6), 823–832.

Catani, M., & De Schotten, M. T. (2008). A diffusion tensor imaging tractography atlas for virtual in vivo dissections. *cortex*, *44*(8), 1105–1132.

Guevara, P., Duclap, D., Poupon, C., Marrakchi-Kacem, L., Fillard, P., Le Bihan, D., Leboyer, M., Houenou, J., & Mangin, J.-F. (2012). Automatic fiber bundle segmentation in massive tractography datasets using a multi-subject bundle atlas. *Neuroimage*, *61*(4), 1083–1099.

Herlin, B., Uszynski, I., Chauvel, M., Dupont, S., & Poupon, C. (2024). Sex-related variability of white matter tracts in the whole hcp cohort. *Brain Structure and Function*, *229*(7), 1713–1735.

La Corte, E., Eldahaby, D., Greco, E., Aquino, D., Bertolini, G., Levi, V., Ottenhausen, M., Demichelis, G., Romito, L. M., Acerbi, F., Broggi, M., Schiariti, M. P., Ferroli, P., Bruzzone, M. G., & Serrao, G. (2021). The frontal aslant tract: A systematic review for neurosurgical applications. *Frontiers in Neurology*, *12*, 641586.
